# The Receptor Kinases DRUS1 and DRUS2 Behave Distinctly in Osmotic Stress Tolerance by Modulating the Root System Architecture via Auxin Signaling

**DOI:** 10.3390/plants13060860

**Published:** 2024-03-16

**Authors:** Ammara Latif, Chen-Guang Yang, Lan-Xin Zhang, Xin-Yu Yang, Xin-Ye Liu, Lian-Feng Ai, Ali Noman, Cui-Xia Pu, Ying Sun

**Affiliations:** 1 Ministry of Education Key Laboratory of Molecular and Cellular Biology, Hebei Research Center of the Basic Discipline of Cell Biology, Hebei Collaboration Innovation Center for Cell Signaling and Environmental Adaptation, Hebei Key Laboratory of Molecular and Cellular Biology, College of Life Sciences, Hebei Normal University, Shijiazhuang 050024, China; ammaralatif94@gmail.com (A.L.);; 2 Department of Botany, Government College University, Faisalabad 38000, Pakistan; 3 Technology Center of Shijiazhuang Customs, Shijiazhuang 050051, China

**Keywords:** osmotic stress (OS), CrRLK1Ls, DRUS1/2, rice, auxin

## Abstract

Receptor kinases *DRUS1* (*Dwarf and Runtish Spikelet1*) and *DRUS2* are orthologues of the renowned *Arabidopsis thaliana* gene *FERONIA*, which play redundant roles in rice growth and development. Whether the two duplicated genes perform distinct functions in response to environmental stress is largely unknown. Here, we found that osmotic stress (OS) and ABA increased *DRUS1* expression while decreasing *DRUS2*. When subjected to osmotic stress, the increased *DRUS1* in *drus2* mutants suppresses the *OsIAA* repressors, resulting in a robust root system with an increased number of adventitious and lateral roots as well as elongated primary, adventitious, and lateral roots, conferring OS tolerance. In contrast, the decreased *DRUS2* in *drus1-1* mutants are not sufficient to suppress *OsIAA* repressors, leading to a feeble root system with fewer adventitious and lateral roots and hindering seminal root growth, rendering OS intolerance. All these findings offer valuable insights into the biological significance of the duplication of two homologous genes in rice, wherein, if one is impaired, the other one is able to continue auxin-signaling-mediated root growth and development to favor resilience to environmental stress, such as water shortage.

## 1. Introduction

Receptor-like kinases (RLKs) are molecular sensors that reside mainly on the cell surface and perform critical functions in plant growth and development under both optimal and stress conditions [1]. RLKs recognize the various signaling molecules, either endogenous or induced clues from environmental fluctuations, through their diverse extracellular domains (ECDs), and then relay the signals by phosphorylating and activating the downstream components via their conserved intracellular domains (ICDs) to trigger a specific biological response [2]. Among these, the *Catharanthus roseus* receptor-like kinase 1-like (CrRLK1L) subfamily stands out, featuring 1~2 malactin domains within their ECDs, comprising 17 members in Arabidopsis and 20 members in rice [1]. In Arabidopsis, nearly all of the CrRLK1Ls have been characterized and are involved in many aspects of plant growth, development, and response to biotic or abiotic stress [2]. Among them, FERONIA (FER) has been extensively studied for its roles in sexual reproduction [3], hypocotyl cell elongation [4], root hair growth under salt stress [5], and pathogen defense [6], etc., by cooperating with other CrRLK1Ls or by itself.

In rice, only a few CrRLK1Ls have been characterized for their biological function. *DWARF AND RUNTISH SPIKELET (DRUS)1/FERONIA-like receptor (FLR)1* and *DRUS2/FLR2* are two orthologues of *FER* that redundantly control stem elongation, inflorescence development, and spikelet fertility [7]. Their roles in male–female interaction, like that of *FER*, are not found yet, but they have been shown to participate in rice–*Magnaporthe oryzae* interaction, together with *FLR11* and *FLR13*, with a different mechanism [8]. *RUPTURED POLLEN TUBE (RUPO)* and male-gene transfer defective 2 (OsMTD2) are essential to maintain the integrity of pollen tubes [9], like that of *ANXUR* (ANX)1 and *ANX2* in *Arabidopsis*. Some other *FLRs* are involved in seed size control [10] and grain quantity regulation [11].

It is interesting that there is only one *FER* gene in Arabidopsis, while two homologous genes of *FER* (*DRUS1* and *DRUS2*) are evolved in rice. In the ideal growth conditions, the presence of either *DRUS1* or *DRUS2* is sufficient to serve the whole life cycle of rice plants and to generate enough progenies, as evidenced by the mostly similar behavior of *drus1-1* and *drus2* single mutants with that of the wild-type [7]. There must exist some logical reasons to maintain these two close homologs with such similar developmental functions. We hypothesized that, if one of them is impaired in function under biotic or abiotic stress conditions, the existence of the other will guarantee plant survival and success in seed setting, since the absence of both *DRUS1* and *DRUS2* will be a disaster to rice growth, which is extremely slow with no seed setting [7]. The opposite roles *DRUS1* and *DRUS2* played in the rice–*Magnaporthe oryzae* interaction give strong support to our hypothesis [8]. Whether these two genes also play different roles in abiotic stress tolerance has not yet been characterized and is an intriguing question to us. The answer to this query will provide some valuable insights into the biological importance of keeping two closely related genes in the rice genome.

Water shortage is a common environmental stress that prompts sessile plants to remodel their root systems to search for heterogeneously distributed water and nutrients [12]. The root system in rice mainly comprises seminal roots (SR), adventitious roots (AR), and lateral roots (LR). Although different genotypes respond differently to osmotic stress, OS-resistant varieties frequently exhibit deep and highly branched root systems in contrast to sensitive ones [13,14]. LRs contribute the most to the total root volume along with ARs and are functionally active components of the root system in terms of water intake [15]. Thus, identification of the key regulators that are capable of increasing the length and lateral roots during osmotic stress will be valuable for improving the OS-tolerance of rice cultivars to guarantee agricultural productivity.

The phytohormones abscisic acid (ABA) and auxin play an essential role in shaping root architecture during water deficits [16]. ABA represses lateral root development by blocking the radial movement of auxin from outer to inner layers during transient water deficits in monocot and eudicot plants [17]. When rice plants encounter compact soil, ABA increases auxin biosynthesis to inhibit primary root elongation and promote root swelling [18]. Auxin governs root growth by balancing cell division and cell elongation in a concentration dependent manner [19]. Changes in auxin local concentration and distribution, via affecting auxin biosynthesis and auxin polar transport during water deficits [20], will modulate this balance, allowing the root to modify its growth patterns based on water availability [21], for instance, to instigate the initiation and elongation of LR in regions with relatively higher water content, resulting in the development of a robust root system [22]. To generate auxin-prompted growth responses, auxin signaling is initiated upon perception by TIR1/AFB receptors, which promote the degradation of Aux/IAA repressor proteins, permitting the release of Auxin Response Factors (ARFs), which ultimately activate the auxin response genes [23].

In order to elucidate the distinct roles of *DRUS1* and *DRUS2* in rice growth, we compared the tolerance of *drus1-1*, *drus2* single mutants, and DJ (wild-type) seedlings to abiotic environmental stress. We discovered that osmotic stress may be the catalyst for the divergent behavior of *DRUS1* and *DRUS2*. *DRUS1* may have a unique role in the OS adaptation of rice plants by generating a more abundant root system while *DRUS2* does not. Our study also explored the behavior of auxin-related genes during OS, demonstrating a significant correlation between OS, *DRUS1*, *DRUS2*, and auxin signaling in rice roots.

## 2. Results

### 2.1. drus1-1 and drus2 Mutants Displayed Distinct Tolerance to Osmotic Stress

**Figure 1:**
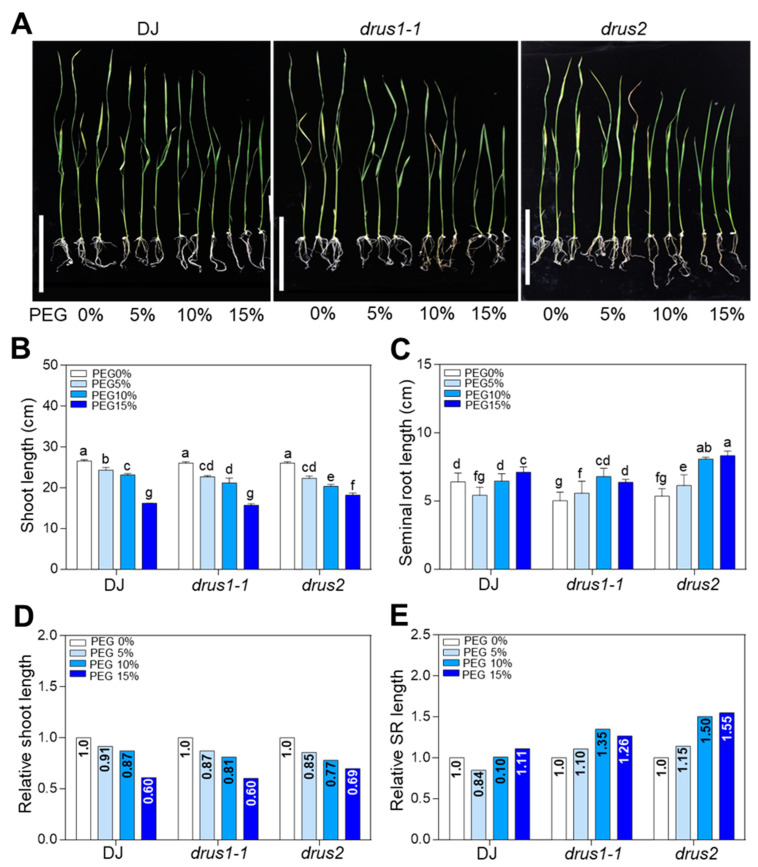
The effect of varying degrees of osmotic stress on seedling growth. (**A**) Seedlings 3 days after germination (DAG) were grown on increasing concentrations of PEG medium for two weeks and photographed. This long-term treatment was performed in the same way as in the following experiments. Bar = 10 cm. (**B**,**C**) Measurement of shoot (**B**) and seminal root (**C**) lengths from seedlings in (**A**). Error bars present means ± SD (n = 15) of three biological replicates; a, b, c, d, e, f, and g indicate the significant differences obtained via two-way ANOVA (Tukey’s test) where *p* < 0.05. (**D**,**E**) Relative shoot (**D**) and root (**E**) length by normalizing with 0% PEG in each genotype.

**Figure 2:**
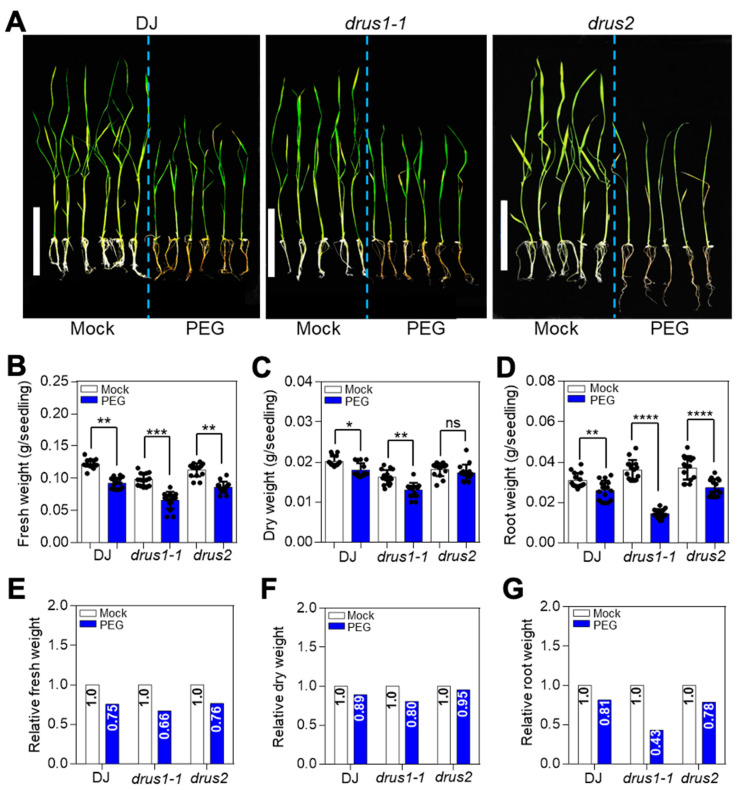
Effect of severe osmotic stress (PEG15%) on seedling biomass. (**A**) Comparative leaf and root phenotype in two-week stressed and non-stressed seedlings. Bar = 10 cm. (**B**,**C**) Measurement of fresh (**B**) and dry (**C**) weight of seedlings in (**A**). (**D**) Measurement of fresh root weight. (**E**–**G**) Relative weight of fresh seedlings (**E**), dry seedlings (**F**), and roots (**G**) after PEG treatment by normalizing with mock in each genotype. Error bars indicate the means ± SD (n = 15) of three biological replicates. Asterisks specify a significant difference, whereas “ns” denotes non-significant, obtain via two-way ANOVA (Tukey’s test), where **** *p* < 0.0001, *** *p* < 0.001, ** *p* < 0.01, and * *p* < 0.05.

Light, temperature, and water supply are three main factors that affect plant growth. To determine the distinct role of *DRUS1* (*Os03g21540*) and *DRUS2* (*Os01g56330*) during environmental stress, *drus1-1* and *drus2* single knockout mutants (T-DNA insertion line in Dongjin [DJ] cultivar from POSTECH RISD [http://signal.salk.edu/cgi-bin/RiceGE], accessed on 12 March 2024) [7] were first treated with darkness, cold (4 °C), and PEG-6000, which is an efficient osmotic stress inducer owing to its high molecular weight and impermeability into plasma membranes [24]—it has proximate osmotic potentials of about −0.19, −0.36, and −0.58 MPa at 10%, 15%, and 20% concentration, respectively [25]. Growth phenotypes with and without treatment revealed that 10-day dark treatment and 10-day cold treatment, respectively, caused a little increase (Supplemental Figure S1A,B) and a great decrease (Supplemental Figure S2A,B) in shoot length in both *drus1-1* and *drus2* mutants, while the root lengths of *drus1-1* and *drus2* remained unchanged, with negligible fluctuations of less than 2% (Supplemental Figures S1C and S2C). Nevertheless, a two-week PEG treatment resulted in obvious modifications in *drus1-1* and *drus2* in a dosage-dependent manner (Figure 1). As the PEG concentration increased, the shoot length steadily decreased, but the root length increased. Notably, at 15% PEG concentration, the *drus2* mutant performed better, exhibiting longer shoot and root lengths compared to DJ. In contrast, the *drus1-1* mutant displayed similar shoot lengths to DJ but had shorter roots (Figure 1A–C). These results preliminary indicate that the *drus2* mutant experiences less shoot growth inhibition (by 31%) and more root elongation promotion (by 55%), while the *drus1-1* mutant experiences more shoot growth inhibition (by 40%) and less root elongation promotion (by 26%) under 15% PEG treatment (Figure 1D,E). Further investigation of biomass accumulation (Figure 2A) with or without treatment revealed a smaller reduction in fresh weight (by 24%), dry weight (by 5%), and root weight (by 22%) in *drus2* seedlings, which was comparable to DJ, while a significant decrease in fresh weight (by 34%), dry weight (by 20%), and root weight (by 57%) occurred in *drus1-1* seedlings, which was not comparable to DJ (Figure 2B–G). These findings suggest that *drus2* seedlings exhibit a degree of tolerance, but *drus1-1* seedlings display heightened sensitivity to 15% PEG treatment.

A number of studies have also employed PEG-6000 in 15% concentration to mimic osmotic stress in vitro [26]. Thus, 15% PEG was used to stimulate osmotic stress (OS) in the subsequent experiments.

### 2.2. drus2 but Not drus1-1 Seedlings Displayed a Strong Root System under OS

**Figure 3:**
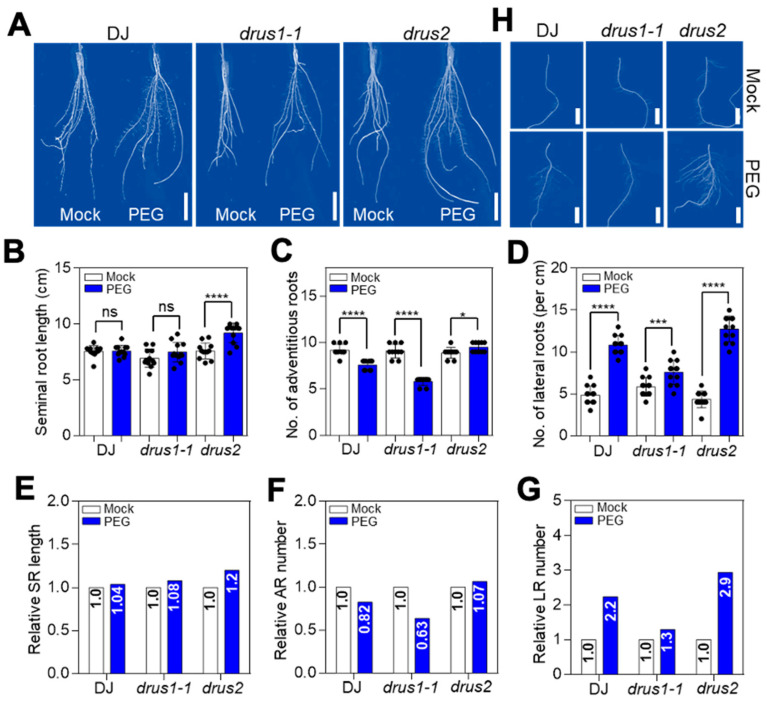
Comparison of root system architecture with and without PEG treatment. (**A**) Morphological features of roots. (**B**–**D**) The length of seminal roots (**B**), the number of adventitious roots (**C**), and number of lateral roots (**D**) with and without two weeks of osmotic stress. (**E**–**G**) Relative seminal root length (**E**), number of adventitious roots (**F**), and number of lateral roots (**G**) after osmotic stress, normalized for each mock. (**H**) The morphology of lateral roots from one representative main root. Bar = 2 cm in (**A**) and 1 cm in (**H**). Error bars in (**B**–**D**) indicate the means ± SD (n = 12) of three biological replicates. Asterisks specify a significant difference, whereas “ns” denotes non-significant, obtained via two-way ANOVA (Tukey’s test), where * *p* < 0.05, *** *p* < 0.005 and **** *p* < 0.001. Abbreviations: SR (seminal root), AR (adventitious root), and LR (lateral root).

The root weight discrepancy between *drus1-1* and *drus2* highlights the different ways in which these two mutants’ roots respond during OS. We then compared the root architecture with or without two-week OS treatment. The *drus2* roots showed a substantial increase in their seminal root length (20%), adventitious root number (7%), and lateral root number (190%), respectively, after treatment, which was much higher than that in DJ (4%, −18%, and 120% increase, respectively) and in *drus1-1* (8%, −27%, and 30% increase, respectively) (Figure 3A–G). Moreover, in *drus2*, not only did the number of LR increase but their lengths also considerably expanded (Figure 3H). In addition, similar results have been obtained in water-shortage soils where the *drus2* mutant, again, presented a highly developed root system as compared to the normal watered soil, while this OS-induced, highly branched and expanded root system was not observed in *drus1-1* and DJ (Supplemental Figure S3). These findings suggest that *DRUS1* and *DRUS2* play distinct roles in shaping root architecture during OS.

### 2.3. DRUS1 but Not DRUS2 Is Highly Induced in the Root by OS at Both the mRNA Level and the Protein Level

**Figure 4:**
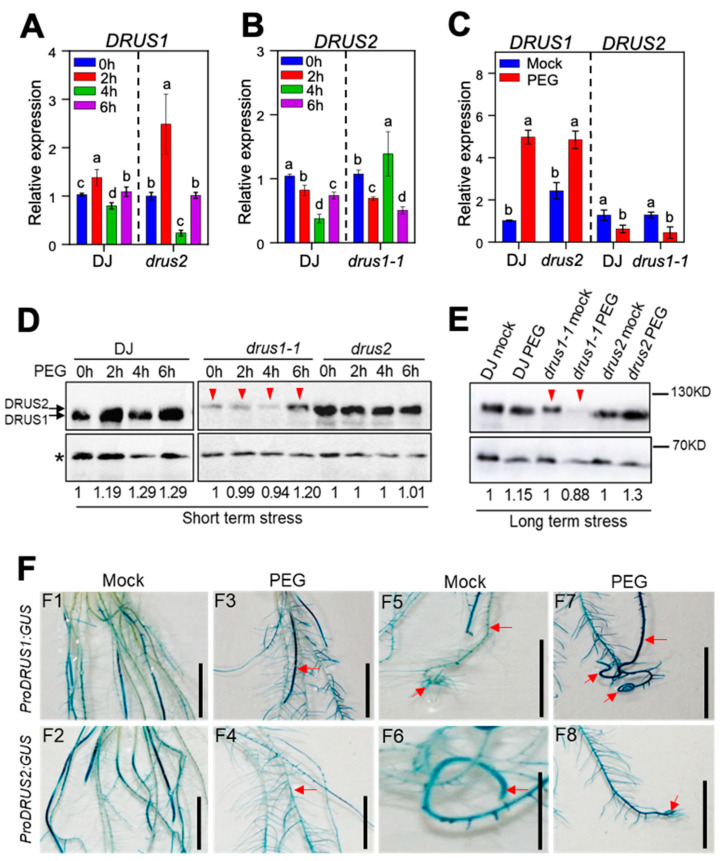
The expression of DRUS1 and DRUS2 in roots in response to osmotic stress (15% PEG). (**A**,**B**) The effects of short-term OS on DRUS1 (**A**) and DRUS2 (**B**) mRNA levels. Two-week-old seedlings treated with PEG for the indicated hours were used to detect mRNA via qRT-PCR. The mRNA level of 0 h in DJ was set to 1. (**C**) The effects of long-term OS on *DRUS1* and *DRUS2* mRNA levels. Seedlings of 3 DAG, treated with PEG or mock for two-weeks, were used to detect mRNA via qRT-PCR. The mRNA level in DJ mock was set to 1. *OsActin* was used as an internal control. Three biological replicates were included for each treatment. a, b, c, and d indicated the significant differences via a one-way ANOVA, where *p* < 0.05. (**D**,**E**) Protein levels of *DRUS1* and *DRUS2* in roots for short- (**D**) and long-term (**E**) osmotic stress. An asterisk marks the non-specific band, while arrows indicate the specific band. Numbers indicate the protein level relative to 0 h stress in DJ, *drus1-1*, and *drus2* mutants after normalizing to a non-specific band. (**F**) Promoter activity analysis of *DRUS1* and *DRUS2* using *ProDRUS1:GUS* and *ProDRUS2:GUS* reporter transgenic plants. Seedlings of 3 DAG, with and without osmotic stress for 14 days, were used for GUS staining. Bar = 1.5 cm. Arrows indicate enhanced or repressed staining.

In order to elucidate the molecular mechanism behind the diverse roles of *DRUS1* and *DRUS2* during OS, the expression patterns of these two genes in three genotypes were investigated. The results disclosed that, in response to short-term OS, the mRNA level of *DRUS1* in DJ and *drus2* oscillated—upregulated at 2 h, somewhat downregulated at 4 h, and, finally, stable at 6 h treatment (4A)—while the mRNA level of *DRUS2* in DJ and *drus1-1* was significantly downregulated in general, though it was slightly upregulated at 4 h treatment in *drus1-1* (Figure 4B). Captivatingly, during long-term (14-day) OS, a sharp increase in *DRUS1* mRNA levels in DJ as well as in *drus2* mutant was observed, whereas a dramatic decrease in *DRUS2* mRNA levels, both in DJ and the *drus1-1* mutant, was observed (4C).

The protein levels of DRUS1 and DRUS2 in root tissues subjected to short-term (2–6 h) OS were also examined using anti-DRUS1 antibodies, which can also recognize DRUS2 [7]. The findings demonstrated that, during the course of treatment, the total protein levels of DRUS1 and DRUS2 in DJ slightly increased (Figure 4D, left panel); the DRUS1 protein level in *drus2* mutant stayed stable, while the DRUS2 protein level in *drus1-1* was significantly lower than DRUS1 in *drus2*, with a modest increase at 6 h of treatment (Figure 4D, right panel). Moreover, after long-term (14-day) OS treatment, noteworthy increases in total DRUS1 and DRUS2 protein levels in DJ and an increase in the DRUS1 protein level in *drus2* mutant were apparent, whereas the DRUS2 protein level in the *drus1-1* mutant marked a great decrease as compared to non-treated one (Figure 4E).

In order to precisely determine the tissue-specific expression of *DRUS1* and *DRUS2*, *proDRUS1:GUS* and *proDRUS2:GUS* reporter lines were used. In general, GUS staining without stress exposure indicates that both genes express in all parts of the roots, including ARs and LRs (Figure 4F(F1,F2)). After 14-day OS exposure, the intensity of GUS staining became stronger in the *proDRUS1:GUS* line but weaker in the *proDRUS2:GUS* line (Figure 4F(F3,F4)). A close view revealed noticeably enhanced GUS staining in the main root, lateral roots, and root tip in the OS-treated *proDRUS1:GUS* line when compared to the non-treated ones (Figure 4F(F5,F7)). However, this kind of enhancement was not found in the *proDRUS2:GUS* line (Figure 4F(F6,F8)). Furthermore, after exposure to 20% PEG for 7 days, the intensity of GUS staining was even increased in the *proDRUS1:GUS* line but sharply decreased in the *proDRUS2:GUS* line, especially in the root tip region (Supplemental Figure S4). These results suggest that OS can activate the *DRUS1* promotor, which is compatible with the highly induced mRNA and protein expression of *DRUS1*, while the *DRUS2* promoter is less active in OS, resulting in a low level of mRNA and protein. Thus, root growth promotion in the *drus2* mutant (Figure 3A, right panel) can be attributed to enhanced *DRUS1* expression, while limited root growth in the *drus1-1* mutant (Figure 3A, middle panel) may be due to repressed *DRUS2* expression under OS. In DJ, growth promotion in lateral roots under OS (Figure 3A, left panel) may be mainly contributed by *DRUS1*. This was supported when observing *pDRUS1:DRUS1-GFP/dk* complementation plants (Supplemental Figure S5A–D), in which the lateral root number was also increased during 14-day OS treatment as compared with non-treatment, similar to that of DJ (Supplemental Figure S5E–J). The DRUS1-GFP protein remains stable after OS (Supplemental Figure S5K)—the same as the above results.

### 2.4. drus2 but Not drus1-1 Seedlings Are Insensitive to ABA Inhibition

**Figure 5:**
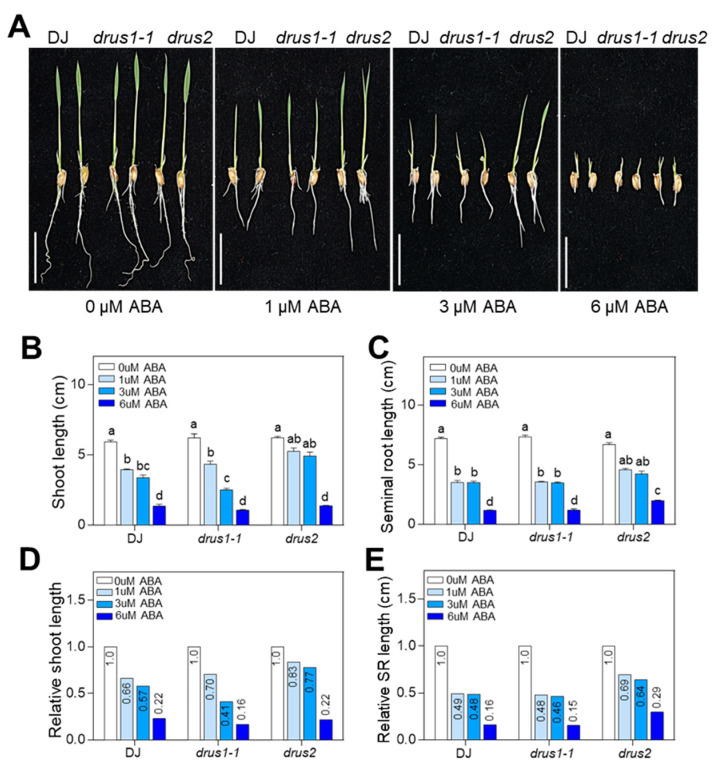
Effect of ABA treatment on root morphology. (**A**) Seedlings of 3 DAG were transferred to Hoagland solution with 0, 1, 3, and 6 µM ABA concentration. After 4 days of treatment, we photographed the seedlings and measured their seminal root lengths. Bar = 2 cm. (**B**–**E**) Statistical analysis of shoot (**B**) and root (**C**) lengths and relative length of shoots (**D**) and roots (**E**) for seedlings in (**A**) by normalizing with each 0 µM control. Three biological replicates were included. a, b, c, and d indicate the significant differences obtained via two-way ANOVA, where *p* < 0.05.

**Figure 6:**
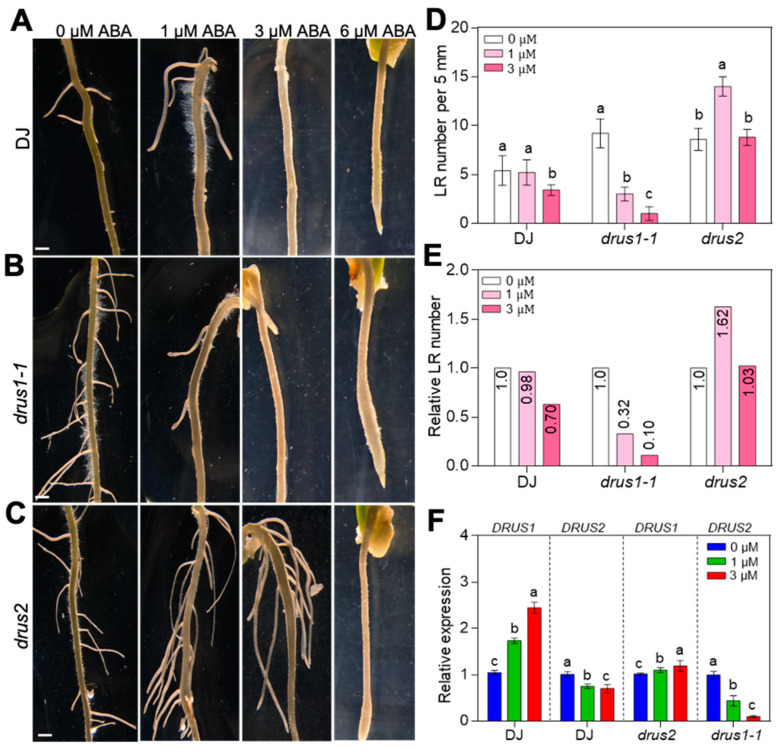
Effect of ABA treatment on lateral root growth. (**A**–**E**) The main root from each seedling in Figure 5A was photographed (**A**–**C**), and the lateral root number (**D**) and relative lateral root number (**E**) to the 0 µm ABA treatment were statistically analyzed. Bar = 2 cm in (**A**–**C**). (**F**) The mRNA levels of *DRUS1* and *DRUS2* in roots in (**A**–**C**) were detected. The mRNA level at 0 µm in DJ was set to 1. Three biological replicates were included. a, b, and c indicated the significant differences obtained via two-way ANOVA, where *p* < 0.05.

DS generally increases ABA biosynthesis and signaling, which repress seedling growth [27]. We next investigated the effects of exogenous ABA on the seedling growth of *drus1-1* and *drus2*. The results showed that the shoot and root growth in *drus2* was less inhibited by 4-day ABA treatment at 1 and 3 µM as compared to DJ, whereas the shoot growth in *drus1-1* was greatly inhibited at 3 and 6 µM ABA as compared to DJ; the root length in *drus1-1* was inhibited similarly to that of DJ (Figure 5A–E). Moreover, with the increase in ABA concentration, less lateral roots developed in *drus1-1* and DJ (Figure 6A,B,D,E), while more lateral roots developed in *drus2* (Figure 6C–E) after 4-days treatment; further, the mRNA level of *DRUS1* in the roots was gradually increased in DJ and *drus2*, but the mRNA level of *DRUS2* was gradually reduced in DJ and *drus1-1* (Figure 6F). These results are consistent with that of PEG treatment, suggesting that *DRUS1* positively regulates root growth under OS, probably in an ABA-dependent manner.

### 2.5. The Root Growth of drus1-1 Is Sensitive But drus2 Is Insensitive to Auxin Deprivation

**Figure 7:**
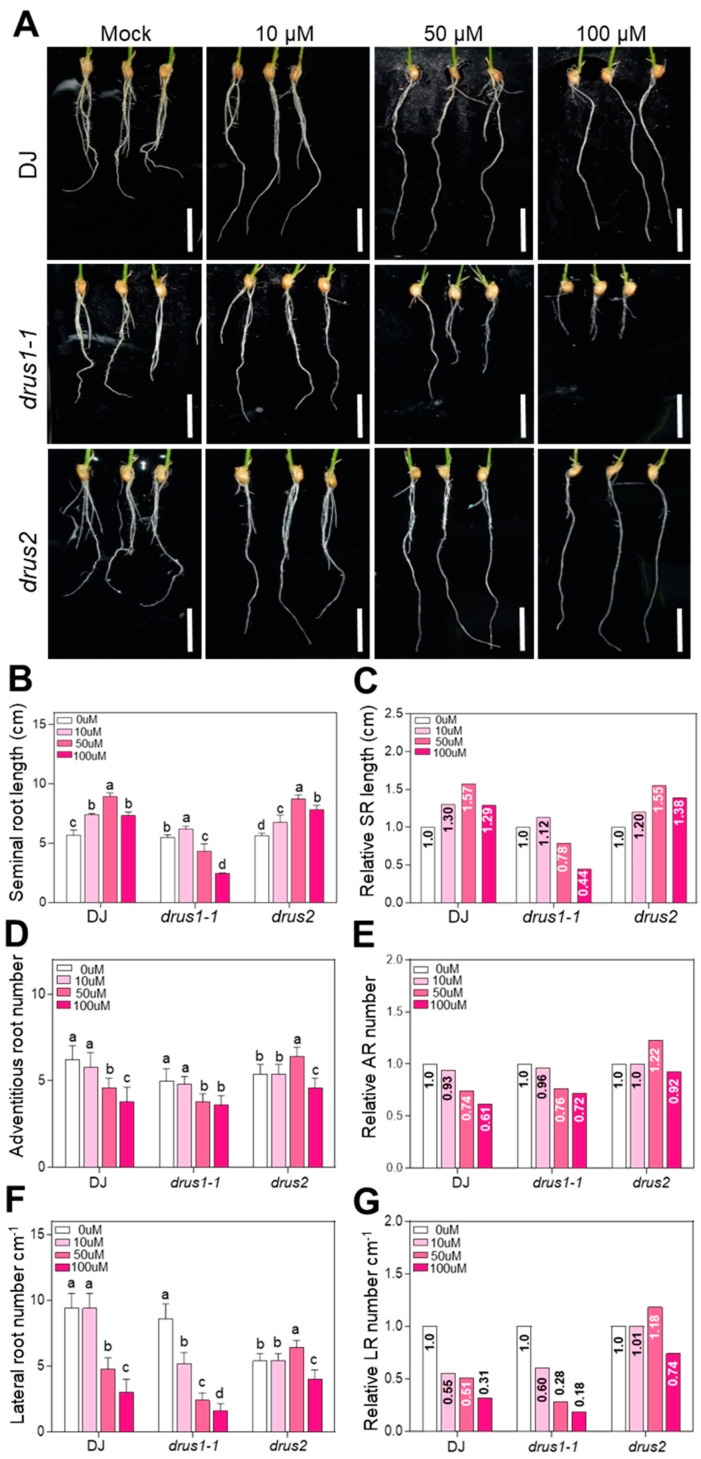
Comparison of the effects of Yucasin on root growth. (**A**) Seedlings of 3 DAG were transferred to a medium containing different concentrations of Yucasin for another 5 days. Bar = 2 cm. (**B**–**G**) Statistical representation of seminal root lengths (**B**), relative SR length (**C**), adventitious root number (**D**), relative AR number (**E**), lateral root number per cm (**F**), and relative LR number per cm (**G**) to 0 µM Yucasin. Data are presented as means ± SD (n = 10), and three biological replicates are included. a, b, c, and d indicate significant differences, where *p* < 0.05 (Tukey’s multiple comparison test). Abbreviations: SR, seminal root; AR, adventitious root; LR, lateral root.

Auxin is a well-known phytohormone controlling root development and the initiation of lateral roots [28]. Since root growth is suppressed during OS, we wondered if the auxin content has changed. Quantification of auxin using HPLC-ESI-MS/MS revealed that the IAA content in the roots of DJ, *drus1-1*, and *drus2* seedlings dramatically declined to a low level after 14-day OS treatment, without significant differences among the three types of plants (Supplemental Figure S6). *YUCCAs* encode auxin biosynthesis genes. The expression of 14 *OsYUCCA* genes in roots with or without 14-day OS treatment was examined. The results showed that some of the *OsYUCCA* genes were upregulated by more than 0.5-fold by PEG treatment: four (*OsYUCCA1*, *-2*, *-3*, and *-7*), one (*OsYUCCA5*), and four (*OsYUCCA1*, *-4*, *-6*, and *-7*) in DJ, *drus1-1*, and *drus2*, respectively. In contrast, a large number of genes were downregulated by PEG treatment: nine (*OsYUCCA5*, *-6*, *-8*, *-9*, *-10*, *-11*, *-12*, *-13*, and *-14*), seven (*OsYUCCA3*, *-4*,*-8*, *-9*, *-10*, *-11*, and *-12*), and six (*OsYUCCA8*, *-10*, *-11*, *-12*, *-13*, and *-14*) in DJ, *drus1-1*, and *drus2*, respectively, while others were not affected much by PEG treatment (Supplemental Figure S7A–N). It seems that these *OsYUCCAs’* mRNA level can hardly correlate with the auxin level: high in mock and low in OS treatment (Supplemental Figure S6H). So, the changes in auxin content via OS may result from the OsYUCCA proteins or enzyme activity. We then used Yucasin, an auxin biosynthesis inhibitor targeting OsYUCCA [29], to reduce the endogenous auxin level and observe the effects on the root growth of three types of plants. The application of 50 and 100 μM Yucasin results in projected inhibition of seminal root growth and more reduced AR and LR numbers in the *drus1-1* mutant while promoting seminal root growth, and less reduced AR and LR numbers in the *drus2* mutant as compared to DJ (Figure 7). Our results indicate that the root growth of *drus1-1* is extremely sensitive, while DJ and *drus2* are insensitive to auxin deprivation. These behaviors of the three types of roots are consistent with those of OS treatment.

### 2.6. The OsIAAs Are Highly Induced in drus1-1 but Not in drus2 Roots

**Figure 8:**
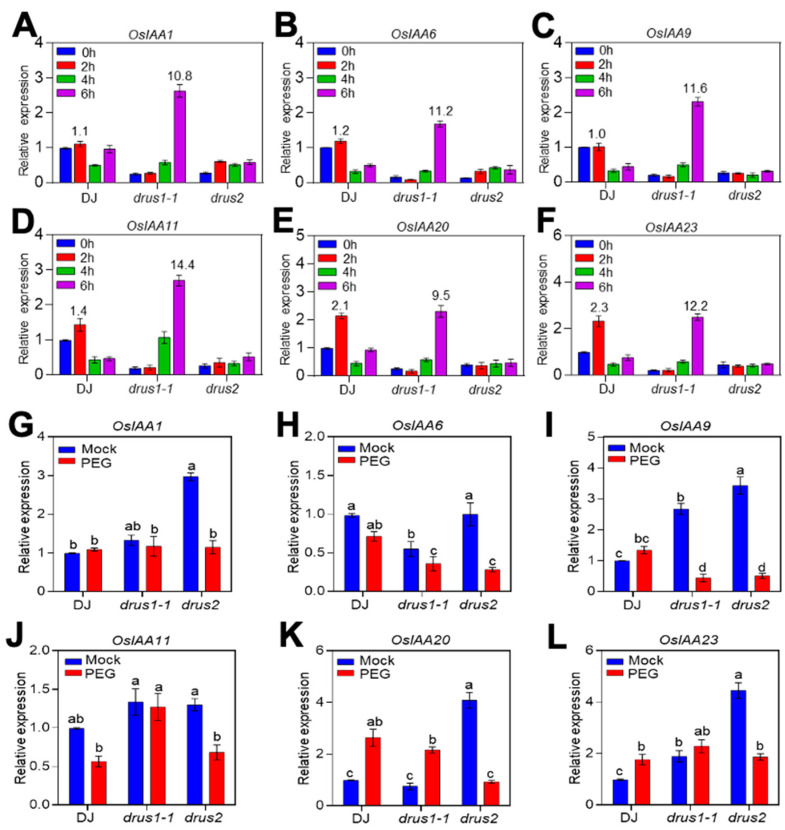
Identification of auxin signaling repressors (*OsIAAs)* in response to OS via RT-qPCR. (**A**–**F**) Seedlings of 14-DAG with short-term OS treatment were used to compare the relative mRNA levels of six *OsIAAs* in the roots. The greater change folds compared to each mock are mentioned on top of the column. (**G**–**L**) Seedlings of 3 DAG with OS treatment for another 14 days were used to compare the relative mRNA level of six *OsIAAs* in the roots. *OsActin* was used as an internal control. The transcript levels in DJ at 0 h (**A**–**F**) or mock (**G**–**L**) were set to 1. On top of the pictures are the names of the genes. The experiment was carried out three times and is presented as the mean ± SD of three biological replicates. a, b, c, and d specify significant differences obtained via two-way ANOVA (Tukey’s test), where *p* < 0.05.

The sensitivity of *drus1-1* roots to Yucasin could not be attributed to auxin deficiency, since a higher content of auxin was found in non-treated *drus1-1* roots (Supplemental Figure S6H). Then, auxin signaling was considered. It involves the degradation of AUX/IAA repressors. Here, we checked *AUX/IAAs*, reported to play significant roles in root development, and found that they behave differentially among the three genotypes before and after OS treatment. In response to short-term OS treatment, the mRNA level of six *OsIAAs* (*OsIAA1*, *-6*, -*9*, -*11*, -*20*, and -*23*) incredibly rose around tenfold or more at 6 h, in contrast to *drus2* and DJ in which these *OsIAAs’* transcript level was maintained at a very low level, although a transient increase at 1 h was observed in DJ (Figure 8A–F). During long-term (14-day) OS treatment, all six *OsIAAs*’ mRNA dramatically decreased in *drus2* compared to non-treated ones; in *drus1-1*, only *OsIAA9* was decreased as much as in *drus2*, while *OsIAA20* was even greatly increased, and four others were maintained at a comparable level to the non-treated ones. In DJ, four *OsIAAs*’ (*OsIAA1*, *-6*, *-20*, and *-23*) expressed similarly to *drus1-1*, *OsIAA11* expressed similarly to *drus2*, and *OsIAA9* expressed differently from *drus1-1* and *drus2.*

Since auxin content declined during OS (Supplemental Figure S6H), *OsIAAs’* expression should accordingly decrease after treatment; that indeed happened in *drus2* but not in *drus1-1* roots due to an unknown factor. We presume that the higher level of *OsIAAs* in *drus1-1* roots would block auxin signaling, while the low level of *OsIAAs* in *drus2* may maintain auxin signaling in active status during OS. These findings collectively elucidate the root phenotypes in three plant types, where, under OS, activated auxin signaling may largely contribute to a well-developed root system in *drus2*, while inactivated auxin signaling is attributed to a poorly developed root system in *drus1-1*.

## 3. Discussion

### 3.1. DRUS1 Plays a Unique Role in the Osmotic Stress Tolerance of Rice Plants

Water deficiency typically limits seedlings’ vigor and leads to a decline in fresh and dry weight; yet, seedlings with improved height and weight under water stress are among the most tolerant ones [30]. Here, we unveiled a distinct function of *DRUS1* from *DRUS2* in OS tolerance. When submitted to OS, DJ (wild-type) and *drus2* mutants possessing the *DRUS1* gene showed a smaller biomass reduction, while the *drus1-1* mutant lacking the *DRUS1* gene showed a greater biomass reduction, as compared to each non-treated rice seedling (Figure 2B–G). The higher biomass accumulation in DJ and *drus2* can attribute to their more expanded root systems, which allow greater intake of water and nutrients in harsh environments. In contrast, the weaker root system, such as smaller SRs and ARs and a smaller number of ARs and LRs, renders *drus1-1* plants more vulnerable to OS (Figure 3).

**Figure 9:**
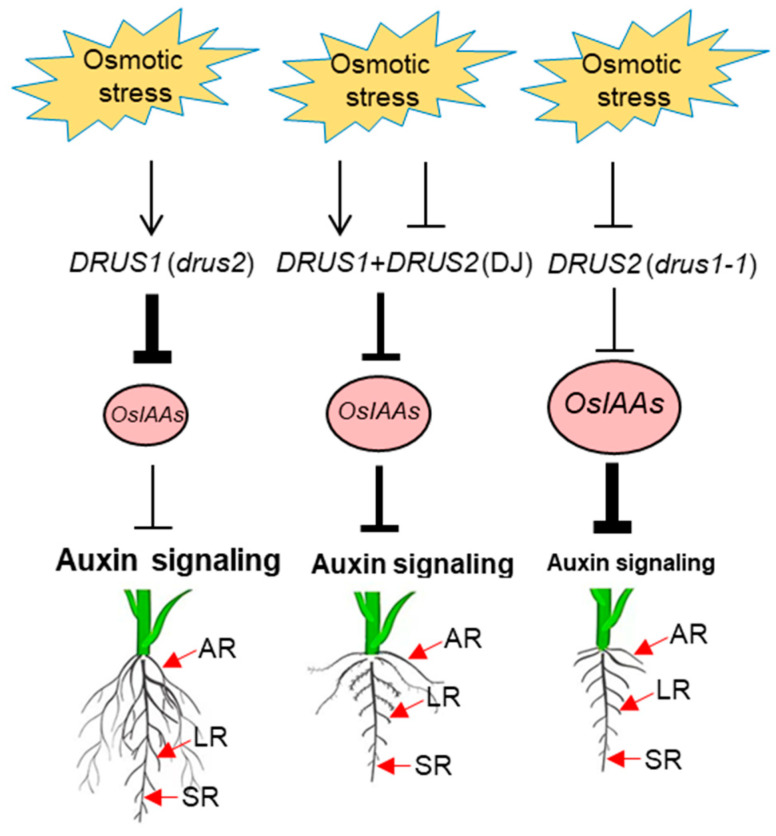
Working model for DRUS1 and DRUS2 under osmotic stress. The DRUS1 function is demonstrated using the *drus2* mutant, and the DRUS2 function is demonstrated using the *drus1-1* mutant. An arrow indicates positive regulation and a line with a dash indicates negative regulation. Thick and thin lines imply more or less inhibition, respectively. A large circle indicates more accumulation of repressors, while a small circle denotes less accumulation. “Auxin signaling” in large size means active and in small size means inactive. SR, seminal root; AR, adventitious root; LR, lateral root.

The enhanced root development in DJ and *drus2*, but not in *drus1-1*, during long-term OS could be resulting from the discrepancy in auxin signaling. Based on all the findings, we proposed that, in *drus2* plants, the *DRUS1* gene was stimulated by OS, and the highly expressed *DRUS1* then inhibits *OsIAAs* transcription, leading to active auxin signaling which facilitates AR and LR production and SR elongation; however, in *drus1-1* plants, the *DRUS2* gene was repressed by OS, and the extremely low level of *DRUS2* is not enough to inhibit *OsIAAs* transcription, resulting in inactive auxin signaling which limits AR and LR production and SR elongation. In DJ, *DRUS1* was stimulated while *DRUS2* was inhibited by OS, and its root phenotype is often between *drus1-1* and *drus2* (Figure 9).

The induction of *DRUS1* and repression of *DRUS2* at both the mRNA level and protein level under OS (Figure 4A–E) are most likely conferred by variations in their promoter regions, whose nucleotide sequences exhibit only 38.48% identity (Supplemental Figure S8A) and harbor various response elements with different numbers. The top two abundant response elements in two genes’ promoters are ERF and LBD, with sixteen and five in *DRUS1* and seventeen and nine in *DRUS2*, respectively (Supplement Figure S8B,C). It has been known that LBD is related to lateral organ boundary regulation, such as adventitious root generation [31]**,** and ERF involves many growth and stress signaling processes [32]. The interaction of these elements with the corresponding transcription factors should be comprehensive; how that leads to the differential expression of two genes remains to be further elucidated.

### 3.2. DRUS1 Promotes Root Expansion under OS by Repressing OsIAAs and Activating Auxin Signaling

Phytohormones are instrumental in shaping root architecture under water limitations [33], with ABA playing a key role in water stress responses and interacting with auxin to impact root architecture modifications and osmotic resistance [34]. Auxin is crucial here for conspicuous deviations in the root architecture, and usually increased auxin levels confer OS tolerance. For instance, the overexpression of YUC genes in rice leads to the formation of ARs, whose initiation and elongation are regulated by a *YUC-auxin-WOX11* (*WUSCHEL-RELATED HOMEOBOX 11*) module [35]. In Arabidopsis, *YUC1* and *-4* are crucial for AR induction, while *YUC6* overexpression confirms its role in OS tolerance [36]. Overexpressing *YUC8*/*9* demonstrated a lower reduction in plant weight to sustain tissue water [37]. In contrast, the *yuc1 yuc2 yuc6* triple mutant significantly reduce endogenous IAA levels and impaired OS tolerance [38]. Similarly, rice seedlings with the inactivated *YUC* gene *CONSTITUTIVELY WILTED 1 (COW1)* displayed typical wilting phenotypes with a reduced root–shoot ratio, emphasizing their role in water homeostasis during water deficits [39]. In this study, we found well-developed roots in *drus2* mutants under OS, where highly induced *OsYUCCA1*, *-4*, and *-7* (Supplemental Figure S7A,D,G) may be related with AR and LR induction due to the local accumulation of IAA; although, the IAA content in the whole roots is not higher than that in *drus1-1* roots (Supplemental Figure S6H).

Our results also suggest that the deviation in root growth patterns in *drus1-1* from *drus2* under OS may be connected to auxin signaling. The auxin signaling pathway is also important in directing root development and, thus, in osmotic stress tolerance. In this pathway, Aux/IAAs (repressors) interact with ARFs (activators) to imprison the auxin response [40], and this pairing eventually hampers auxin signaling. A study revealed that fifteen IAAs interact with eight out of fourteen integrated ARFs [40], and the interaction between ARF7 and IAA3 mediates hydro patterning in response to water stress in *Arabidopsis thaliana* [41]. Here, we did not find obvious differences in *OsARFs* expression between *drus1-1* and *drus2* mutants (Supplemental Figure S7O–Q) but found intense inductions of *OsIAA-1*, *-6*, *-9*, *-11*, *-20*, and *-23* in *drus1-1* which were not evident in *drus2* following OS (Figure 8A–F), suggesting the auxin response to be declined in *drus1-1* but not in *drus2*. Indeed, overexpressing *OsIAAs* always decreases the auxin response and leads to the repression of ARs and LRs as well as the elongation of SR. *OsIAA1-*overexpressing plants are insensitive to auxin-induced growth of ARs and LRs and to auxin-inhibited SR growth [42]. Similarly, *OsIAA9-*overexpressing plants display fewer ARs and LRs, along with reduced inhibition of root elongation by auxin [43], and *OsIAA4-*overexpressing plants show a reduced auxin response, determining dwarf plants and a reduction in gravity responses [44]. The accumulation of *OsIAA11* [45], *OsIAA13* [46]**,** and *OsIAA23* [47], due to a point mutation in the conserved motif in domain II (which is required for IAAs degradation), severely blocks the initiation of LRs and ARs. However, overexpression of *OsIAA6* and *OsIAA20* improves OS tolerance in rice by regulating auxin biosynthesis-related genes [48,49]. These findings support our point that, under OS, induced *OsIAAs* in *drus1-1* block auxin signaling and then repress LR and AR growth while arrested *OsIAAs* in *drus2* keep auxin signaling active and facilitate LR and AR initiation (Figure 3 and Figure 8). To promote root elongation, the IAA content in the root tip should be reduced, as shown in Yucasin treatment (Figure 7). A lower concentration of IAA will increase the elongation region by blocking cell differentiation, and that contributes greatly to root length [50].

Taken together, remodeling the root system architecture during OS requires dynamic IAA distribution and auxin signaling activity. Our findings illuminate the importance of DRUS1 in regulating auxin-mediated root development to aid in osmotic tolerance.

### 3.3. The Biological Significance of the Presence of DRUS1 and DRUS2 Homologues in Rice Genome

In the CrRLK1L subfamily, *FERONIA* (*FER*) is a well-known multifunctional regulator that controls broad aspects of plant growth and development in response to various abiotic and biotic stresses in *Arabidopsis* [4,5,51]. In rice, there are two orthologues of *FER*, *DRUS1* and *DRUS2*, which share 75% and 95% protein sequence identity in their extracellular and intracellular domains, respectively, and function redundantly throughout their life span under ideal growth conditions [7]. These two genes also share more than 50% and near 80% protein sequence identity with *FER* in extracellular and intracellular domains, respectively, and *DRUS1* can rescue *fer* fertility [7], meaning that their molecular function is the same. One *FER* gene in Arabidopsis may be sufficient for the whole life span of *Arabidopsis*, including the propagation of progenies—if *FER* is damaged or even knocked out, the *fer* mutants, despite the existence of certain developmental abnormalities, retain the ability to set seeds [51]. Nevertheless, the double-knockout mutants *drus1-1* and *drus2* develop poorly and can hardly set seeds. This implies that *DRUS1* and *DRUS2* are important for rice life spans and that a single orthologue is not sufficient to ensure the propagation of progenies in situations where its functionality is compromised by environmental stressors. Here, we demonstrated that the *DRUS2* gene is repressed when subject to water shortage; therefore, the *DRUS1* gene can still function to activate root growth and development. It is also reported that *DRUS1*/*FLR1*, but not *DRUS2*/*FLR2*, functions in M. Oryzae resistance [8]. However, which stressors compromise *DRUS1* remains unknown.

In addition to *DRUS1* and *DRUS2*, another pair of homologues in rice, *OsCrRLK1L10* (*Os05g06990*) and *OsCrRLK1L14* (*Os06g28810*), are also expressed in the roots and triggered by water stress [52], suggesting a significant role of this subfamily in water and nutrients usage efficiency. Recently, *OsMRLK63*, an *OsCrRLK1L* like RLK in rice, has been reported to confer OS tolerance by regulating reactive oxygen species (ROS) production in leaves [53]. Beyond *DRUS1*, which contributes to OS tolerance by endorsing root system development, RLKs from other subfamilies also employ distinct pathways to govern OS adaptation. For instance, *OsSIK1* and *FON1* from the leucine-rich repeat receptor-like kinase (LRR-RLK) subfamily activate the ABA signaling pathway [54] and improve the antioxidant system and limit the stomatal density of leaves to diminish water loss [55], respectively, while *Leaf Panicle 2* (*LP2*) in this subfamily serves as a negative regulator by interacting with drought-responsive aquaporin proteins [56]. *OsSIK2* and *OsESG1* from the S-domain RLK subfamily activate the detoxification of ROS [57] and regulate AR development [58]**,** respectively.

Nowadays, more and more RLK functions are dissected in rice, which are involved not only in plant growth and development but also in responses to diverse environmental challenges, which are much more complicated than those in Arabidopsis, demonstrating the biological significance of the gene duplication of *RLKs* that is almost double in the rice genome as compared to *Arabidopsis* [59].

## 4. Materials and Methods

### 4.1. Plant Materials and Growth Conditions under Osmotic Stress

Wild-type rice (*Oryza sativa* ssp. *japonica*) plants, Dongjin (DJ), *drus1-1* and *drus2* mutants [7], and transgenic plants were grown in a light chamber under 28 °C for 8 h of dark and 16 h of light. For long-term treatment, seeds, after surface sterilization with 2.5% Sodium Hypochlorite solution, were transferred to half MS (Murashige–Skoog) with 0.3% phytagel for three days to allow germination and then shifted to glass bottles containing half MS with and without 15% (*w*/*v*) PEG-6000 (Sigma-Aldrich, Shanghai, China) for two weeks. For short-term treatment, sterilized seeds were grown in Hoagland’s solution for 2 weeks and treated with 15% PEG for 0, 2, 4, and 6 h. The roots were collected and stored at −80 °C until further use.

### 4.2. GUS Staining Assay

*ProDRUS1:GUS* and *ProDRUS2:GUS* transgenic [7] with and without 2 weeks of osmotic stress were prefixed in 90% acetone at −20 °C for 15 min. We washed the samples thrice with PBS, applied staining solution (10 mM Sodium EDTA, 5 mM Potassium ferricyanide, 5 mM potassium Ferro cyanide, 100 mM Sodium Phosphate Buffer with pH 7.0, 0.1 mg chloramphenicol, 0.1% Triton X-100, and 2mM5-bromo-4-chloro-3-indolyl-b-glucuronic acid) for half an hour, and incubated the samples in increasing concentrations of ethanol [60]. A Nikon-D7100 (Nikon, Tokyo, Japan)camera was used to capture the images.

### 4.3. Immunoblot Analysis

Total protein was extracted from the roots of wild-type and *drus1-1* and *drus2* single mutant samples using 2×SDS protein sample buffer, then separated using 6% and 4% SDS-PAGE and transferred to nitrocellulose membrane. The blot was probed with anti-DRUS1 antibodies, which was raised against the *DRUS1* C-terminal that share a 96% identity with the *DRUS2* C-terminal [7], then with anti-rabbit-HRP (Bio-Rad, Shanghai, China) antibody, and imagined by the Western Blotting Analysis System (GE Healthcare, US).

### 4.4. Quantitative Reverse Transcription-PCR (qRT-PCR)

Total RNA was extracted from the roots by using Trizol reagent (Invitrogen, Shanghai, China). A total of 250 ng of total RNA was used as the template for cDNA synthesis using a PrimeScript RT reagent kit with gDNA Eraser (Takara Bio, Beijing, China). qRT-PCR was performed on the Applied Biosystems 7500 (ThermoFisher Scientific, Shanghai, China) using SYBR Green Premix Ex Taq II (Tli RNaseH Plus; TaKaRa Biotechnology, 0427) with a 20 µL sample volume. *OsActin1* was used as an internal control. All the primers used in this study are listed in Supplemental Table S1. The relative expression level of each gene was calculated using the standard ΔΔCT approach. To illustrate all the data, GraphPad prism 5 software was used.

### 4.5. Auxin Content Measurement

About 0.1 g of root tissues was collected from 7-day-old seedlings treated for 10 h with or without PEG, as well as from 2-week-old seedlings (treated or non-treated). Tissues were immediately frozen in liquid nitrogen before being pulverized into a fine powder and extracted using 0.1% formic acid methanol solution. We blow-dried the residue with nitrogen and reconstituted it with 20% methanol: 0.1% formic acid water solution (*v*/*v*). After filtration IAA concentration was analyzed using a HPLC-ESI-MS/MS (Waters Xevo TQ-S; Waters ACOUITY UPLC-MS/MS, UK).

### 4.6. Yucasin Treatment

Hydro-primed seeds for three days were transferred to Hoagland solutions containing 0, 10, 50, and 100 µM Yucasin (5-(4-Chlorophenyl)-2,4-dihydro-[1,2,4]-triazole-3-thione). The root phenotype was inspected and photographed using a camera after five days of treatment. The ImageJ software was used to measure the root lengths. The whole protocol was repeated 3 times. The graphs were created using Graphpad Prism 5 and the significance was determined using two-way ANOVA (Tukey’ test).

## Data Availability

Data are contained within the article and Supplementary Materials.

## Supplementary Materials

The following supporting information can be downloaded at: https://www.mdpi.com/article/10.3390/plants13060860/s1, Figure SS1. Effect of dark treatment on seedlings’ growth. (A) Seedlings of 3 days after germination (DAG) were transferred to a light chamber following 16/8 h of light/dark period at 28 °C, or remained in a dark chamber at 28 °C. After 10 days of treatment the phenotype of all three type of seedlings were compared. Bar = 10 cm (B, C) The relative shoot (B) and root (C) lengths were measured and statistically analyzed, n = 10; Figure S2. Effect of cold stress (4 °C) on seedlings growth. (A) Seedlings of 3 DAGs were transferred to a light chamber at 28 °C or 4 °C, following 16/8 h of light/dark period for 10 days. The phenotype of all three types of seedlings were compared. Bar = 10 cm (B, C) The relatvie shoot (B) and root (C) lengths were measured and statistically analyzed. n = 10; Figure S3. Comparison of root morphology of DJ, drus1-1, and drus2 mutants under soil water stress. Ten-day old seedlings have been stressed or not stressed by a 12-day water shortage, then the roots were cleaned for comparative phenotype. Bar = 4 cm; Figure S4. Promoter activity analysis of DRUS1 and DRUS2 using ProDRUS1:GUS and ProDRUS2:GUS reporter transgenic plants. Seedligns of 3 DAGs were treated with increasing concentrations of PEG for 7 days. The representative roots from each treatment were used for comparative GUS staining. Red arrows show distinct expression in two lines after PEG treatment. Bar = 2.5 cm; Figure S5. *ProDRUS1:DRUS1-GFP/dk* complementation plants increased their lateral root number in response to DS. (A–D) Comparative phenotype of DJ (A, C) and ProDRUS1:DRUS1-GFP/dk (drus1-1 drus2 double knockout) (B, D) seedlings, which were germinated for 3 days and treated (C, D) or non treated (A, B) with PEG for 14 days (Bar = 2 cm). (E–H) The root architecture from the seedlings in (A), (B), (C) and (D), respectively, Bar = 500 μm. (I, J) The lateral root number (I) and relative root number (J) analysed for the roots in (E-H). Error bar in (I) presented as mean ± SD (n = 5) by student T-test, where ** *p* < 0.01, *** *p* < 0.001. (K) The protein expression of DRUS1-GFP in the complementation plants with and without PEG treatment by immunoblot assay. Figure S6. Measurements of IAA content in root of indicated plants by HPLC-ESI-MS/MS. (A–F) The chart of IAA peak before (balck arrows) and after (red arrows) DS treatment. (G) Standard chart with 10 PPM IAA. (H) Bar graphy of IAA content for three types of plant roots. Error bar indicated mean ± SD of three biological replicates; Figure S7. Identification of auxin biosynthesis genes and signaling genes in response to drought stress by qRT-PCR. The relative mRNA level of auxin biosynthetic genes (A–N) and Auxin Response Factor (OsARF) genes (O–Q) in roots from three types of seedlings with or without 14-day drought stress were compared. OsActin was used as an internal control. The transcript level in DJ mock or at 0 h were set to 1. On top of the pictures are the names of the genes. The experiment was carried out three times and presented as the mean ± SD. a, b, and c specified significant differences by two-way ANOVA (Tukey’s test), where *p* < 0.05; Figure S8. Promoter sequence analysis. (A) Alignment of promoter sequence of DRUS1 (2337 bp in length) and DRUS2 (2247 bp in length) which were used to drive GUS reporter. (B, C) Top 30 response elements in promoter region of DRUS1 (B) and DRUS2 (C); Table S1. A list of primers used in qRT-PCR.
