# Supplementary material for: The Receptor Kinases DRUS1 and DRUS2 Behave Distinctly in Osmotic Stress Tolerance by Modulating the Root System Architecture via Auxin Signaling
Source: Plants (Basel). 2024 Mar 16;13(6):860. doi: 10.3390/plants13060860 (PMC10974500; doi:10.3390/plants13060860)
Supplement: Supplementary file 1 [file plants-13-00860-s001.zip › plants-2866115-supplementary.pptx]

## Slide 1
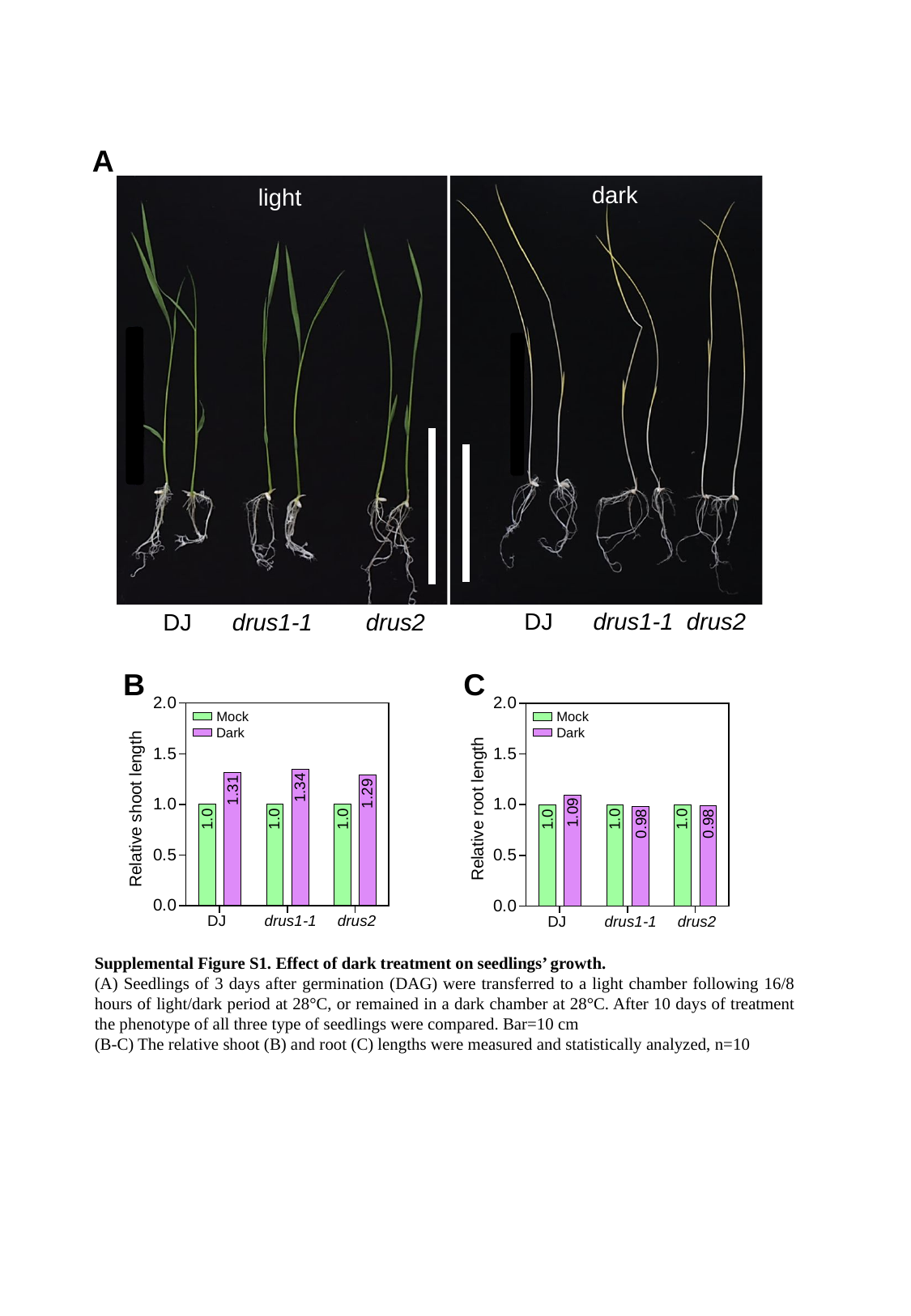

A
dark
 DJ drus1-1 drus2
light
 DJ drus1-1 drus2
B
C
Mock
Dark
Relative shoot length
DJ drus1-1 drus2
Mock
Dark
Relative root length
DJ drus1-1 drus2
1.34
1.31
1.29
1.09
1.0
1.0
1.0
1.0
1.0
1.0
0.98
0.98
Supplemental Figure S1. Effect of dark treatment on seedlings’ growth.
(A) Seedlings of 3 days after germination (DAG) were transferred to a light chamber following 16/8 hours of light/dark period at 28°C, or remained in a dark chamber at 28°C. After 10 days of treatment the phenotype of all three type of seedlings were compared. Bar=10 cm
(B-C) The relative shoot (B) and root (C) lengths were measured and statistically analyzed, n=10

## Slide 2
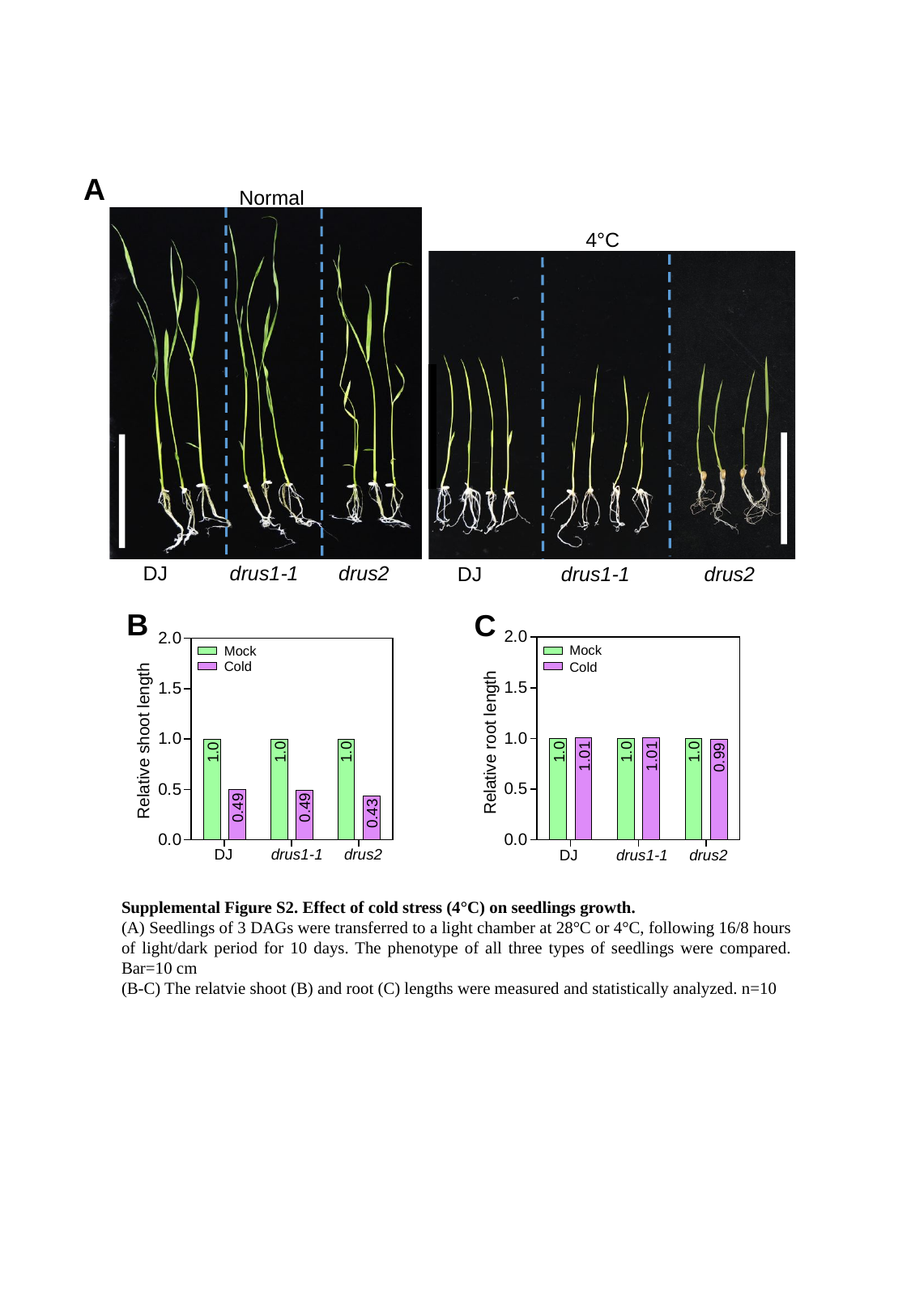

A
Normal
 DJ drus1-1 drus2
4°C
 DJ drus1-1 drus2
B
C
Mock
Cold
Relative root length
DJ drus1-1 drus2
Mock
Cold
Relative shoot length
DJ drus1-1 drus2
1.0
1.0
1.0
1.0
1.0
1.0
1.01
1.01
0.99
0.49
0.49
0.43
Supplemental Figure S2. Effect of cold stress (4°C) on seedlings growth.
(A) Seedlings of 3 DAGs were transferred to a light chamber at 28°C or 4°C, following 16/8 hours of light/dark period for 10 days. The phenotype of all three types of seedlings were compared. Bar=10 cm
(B-C) The relatvie shoot (B) and root (C) lengths were measured and statistically analyzed. n=10

## Slide 3
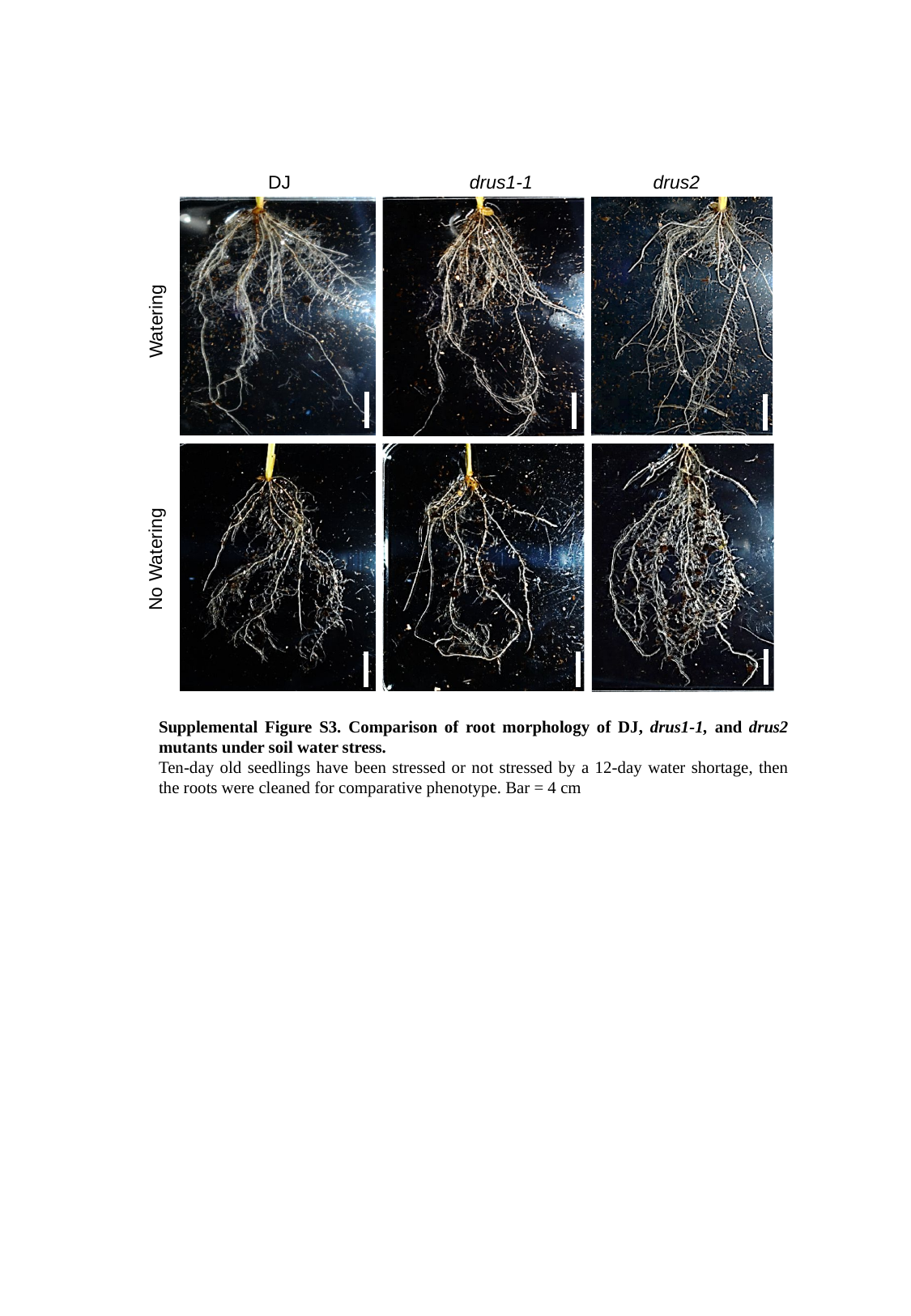

DJ drus1-1	 drus2
Watering
No Watering
Supplemental Figure S3. Comparison of root morphology of DJ, drus1-1, and drus2 mutants under soil water stress.
Ten-day old seedlings have been stressed or not stressed by a 12-day water shortage, then the roots were cleaned for comparative phenotype. Bar = 4 cm

## Slide 4
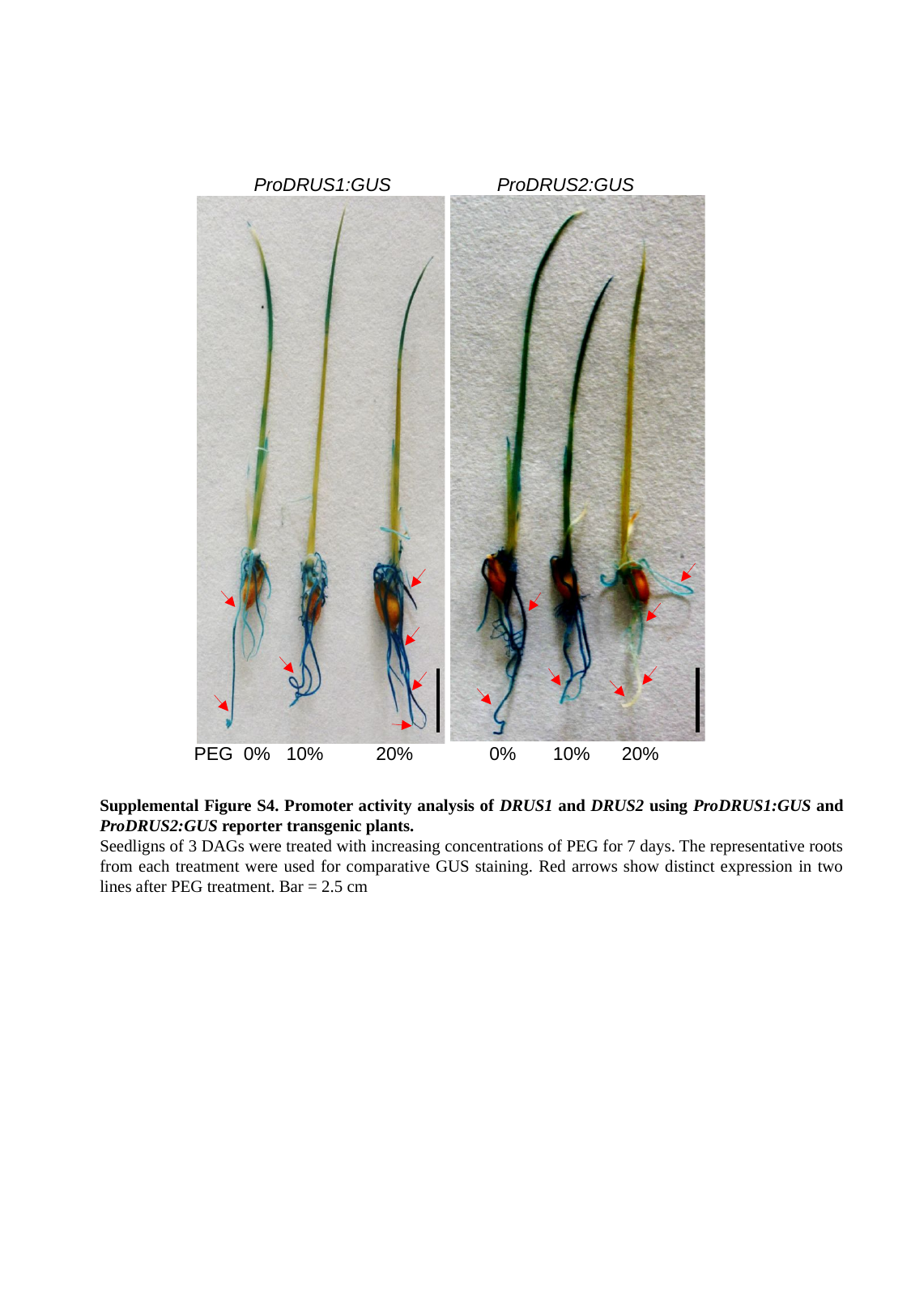

ProDRUS1:GUS
ProDRUS2:GUS
PEG 0% 10% 20%	 0% 10% 20%
Supplemental Figure S4. Promoter activity analysis of DRUS1 and DRUS2 using ProDRUS1:GUS and ProDRUS2:GUS reporter transgenic plants.
Seedligns of 3 DAGs were treated with increasing concentrations of PEG for 7 days. The representative roots from each treatment were used for comparative GUS staining. Red arrows show distinct expression in two lines after PEG treatment. Bar = 2.5 cm

## Slide 5
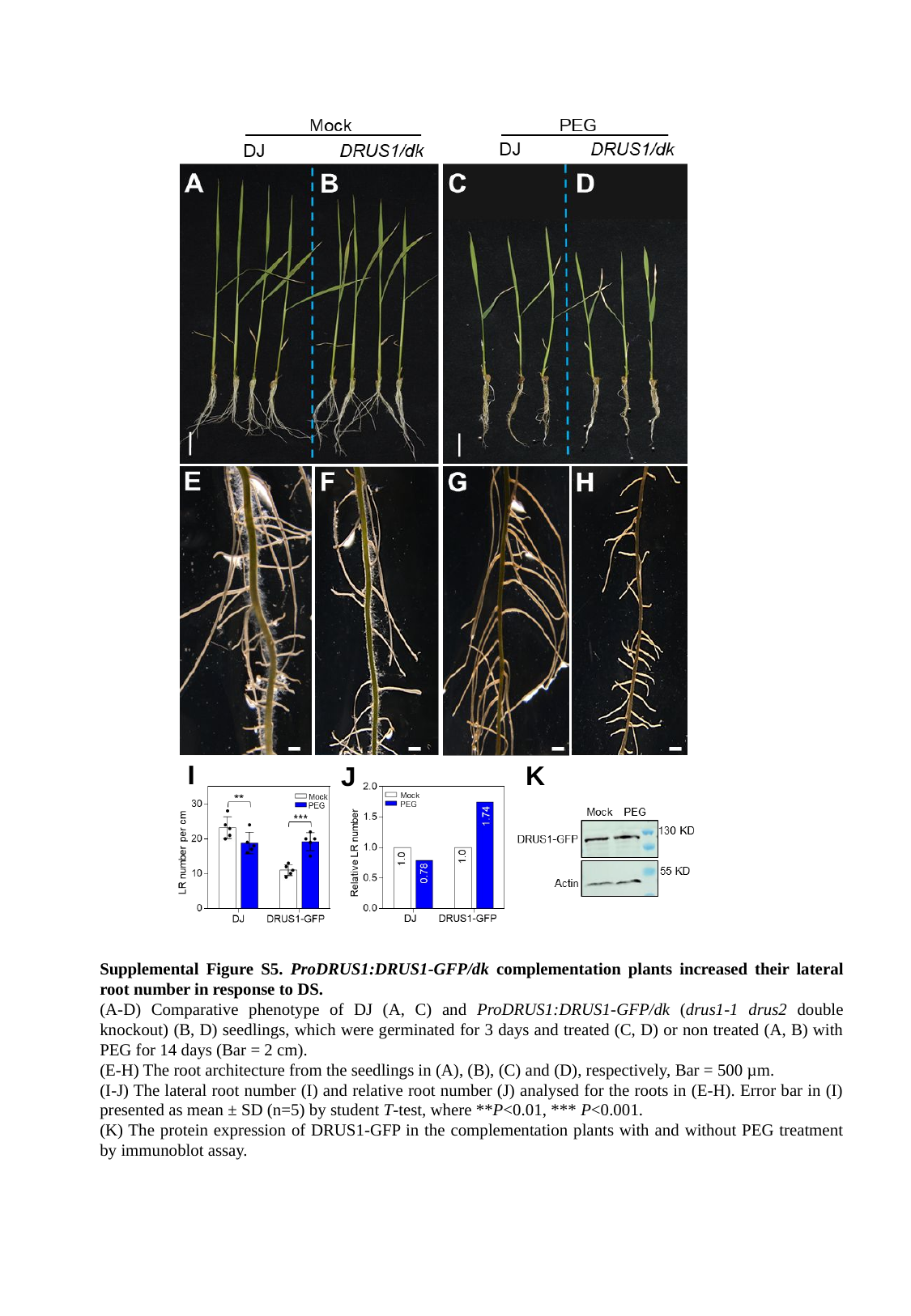

I
K
J
Supplemental Figure S5. ProDRUS1:DRUS1-GFP/dk complementation plants increased their lateral root number in response to DS.
(A-D) Comparative phenotype of DJ (A, C) and ProDRUS1:DRUS1-GFP/dk (drus1-1 drus2 double knockout) (B, D) seedlings, which were germinated for 3 days and treated (C, D) or non treated (A, B) with PEG for 14 days (Bar = 2 cm).
(E-H) The root architecture from the seedlings in (A), (B), (C) and (D), respectively, Bar = 500 µm.
(I-J) The lateral root number (I) and relative root number (J) analysed for the roots in (E-H). Error bar in (I) presented as mean ± SD (n=5) by student T-test, where **P<0.01, *** P<0.001.
(K) The protein expression of DRUS1-GFP in the complementation plants with and without PEG treatment by immunoblot assay.

## Slide 6
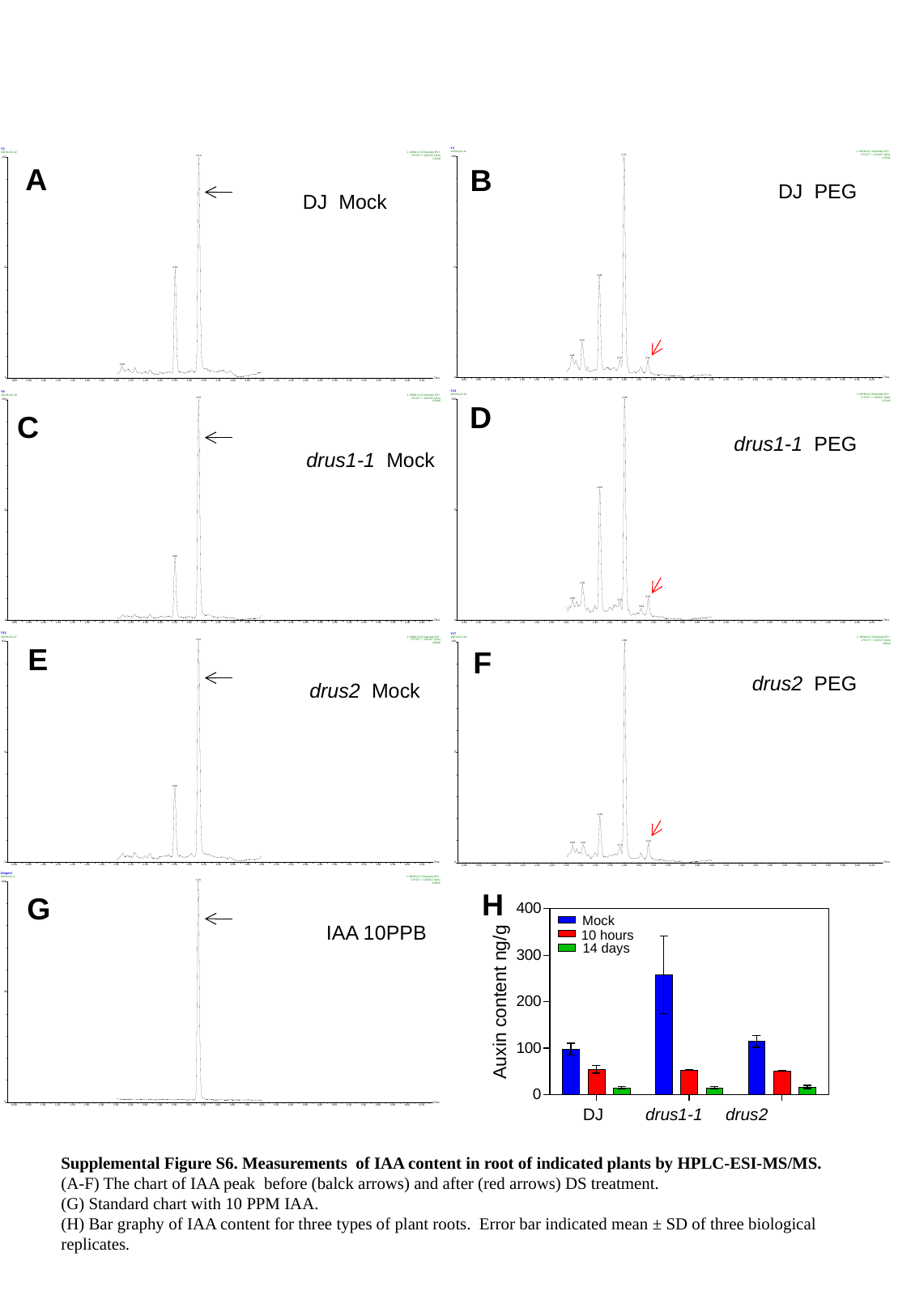

A
B
DJ PEG
DJ Mock
D
C
drus1-1 PEG
drus1-1 Mock
E
F
drus2 PEG
drus2 Mock
H
G
IAA 10PPB
Mock
10 hours
 14 days
Auxin content ng/g
 DJ drus1-1 drus2
Supplemental Figure S6. Measurements of IAA content in root of indicated plants by HPLC-ESI-MS/MS.
(A-F) The chart of IAA peak before (balck arrows) and after (red arrows) DS treatment.
(G) Standard chart with 10 PPM IAA.
(H) Bar graphy of IAA content for three types of plant roots. Error bar indicated mean ± SD of three biological replicates.

## Slide 7
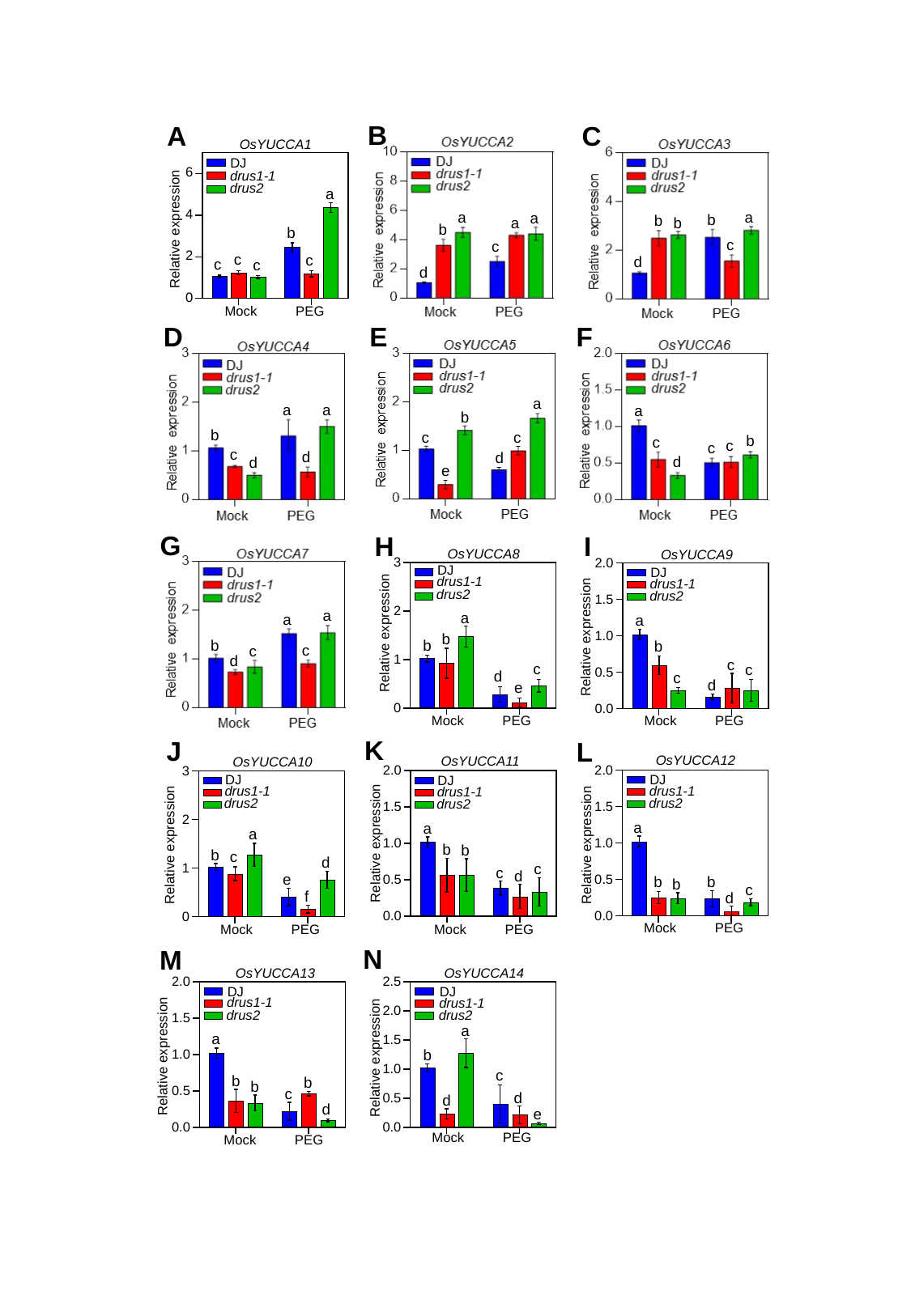

B
a
a
a
b
c
d
A
OsYUCCA1
DJ
drus1-1
drus2
a
Relative expression
b
c
c
c
c
Mock PEG
C
a
b
b
b
c
d
F
a
b
c
c
c
d
D
a
a
b
c
d
d
E
a
b
c
c
d
e
G
a
a
b
c
c
d
H
OsYUCCA8
DJ
drus1-1
drus2
a
Relative expression
b
b
c
d
e
Mock PEG
I
OsYUCCA9
DJ
drus1-1
drus2
a
Relative expression
b
c
c
c
d
Mock PEG
K
OsYUCCA11
DJ
drus1-1
drus2
a
Relative expression
b
b
c
c
d
Mock PEG
J
OsYUCCA10
DJ
drus1-1
drus2
a
Relative expression
b
c
d
e
f
Mock PEG
L
OsYUCCA12
DJ
drus1-1
drus2
a
Relative expression
b
b
b
c
d
Mock PEG
N
OsYUCCA14
DJ
drus1-1
drus2
a
b
Relative expression
c
d
d
e
Mock PEG
M
OsYUCCA13
DJ
drus1-1
drus2
a
Relative expression
b
b
b
c
d
Mock PEG

## Slide 8
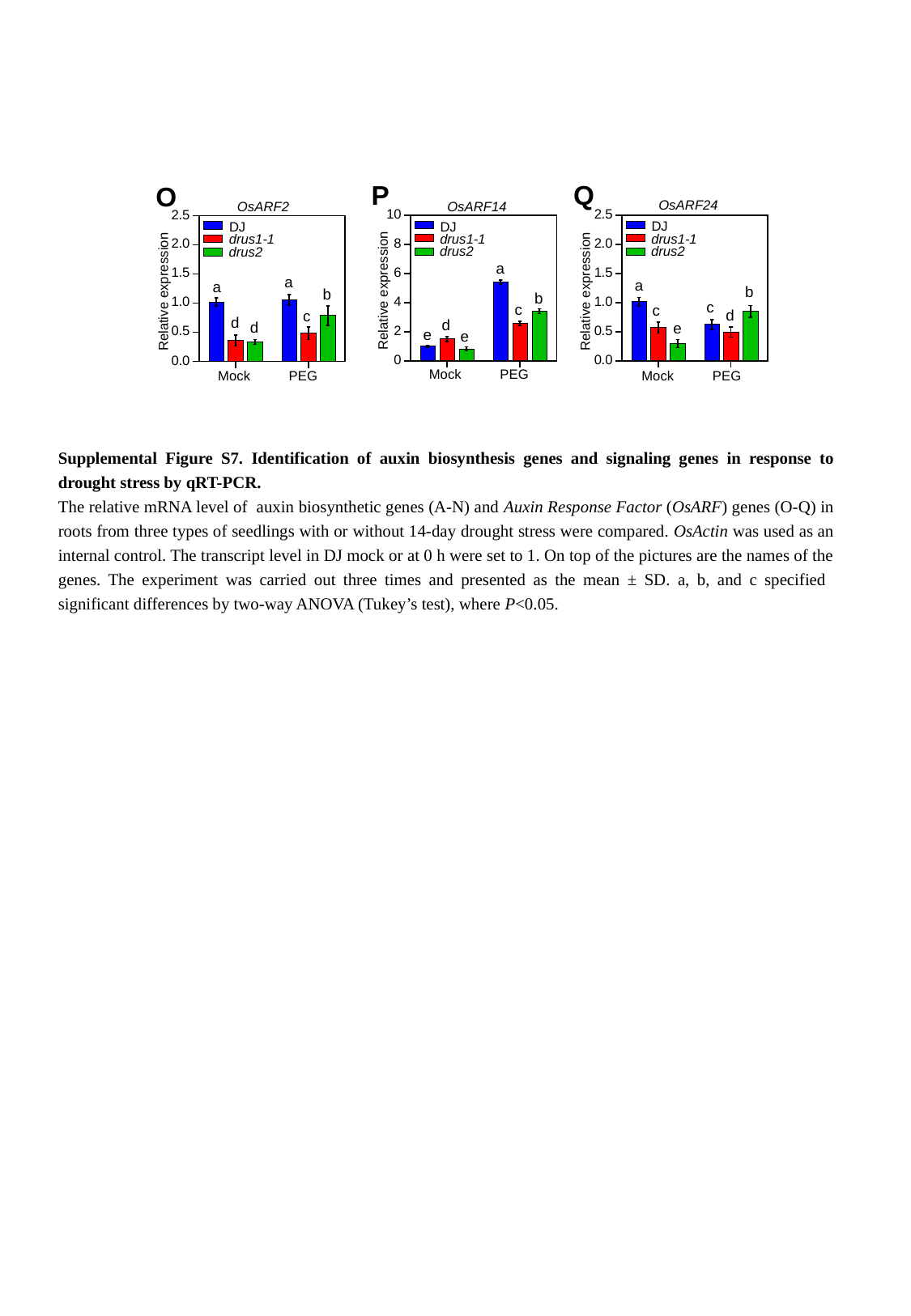

P
OsARF14
DJ
drus1-1
drus2
Relative expression
Mock PEG
a
b
c
d
e
e
Q
OsARF24
DJ
drus1-1
drus2
Relative expression
Mock PEG
a
b
c
c
d
e
O
OsARF2
DJ
drus1-1
drus2
Relative expression
Mock PEG
a
a
b
c
d
d
Supplemental Figure S7. Identification of auxin biosynthesis genes and signaling genes in response to drought stress by qRT-PCR.
The relative mRNA level of auxin biosynthetic genes (A-N) and Auxin Response Factor (OsARF) genes (O-Q) in roots from three types of seedlings with or without 14-day drought stress were compared. OsActin was used as an internal control. The transcript level in DJ mock or at 0 h were set to 1. On top of the pictures are the names of the genes. The experiment was carried out three times and presented as the mean ± SD. a, b, and c specified significant differences by two-way ANOVA (Tukey’s test), where P<0.05.

## Slide 9
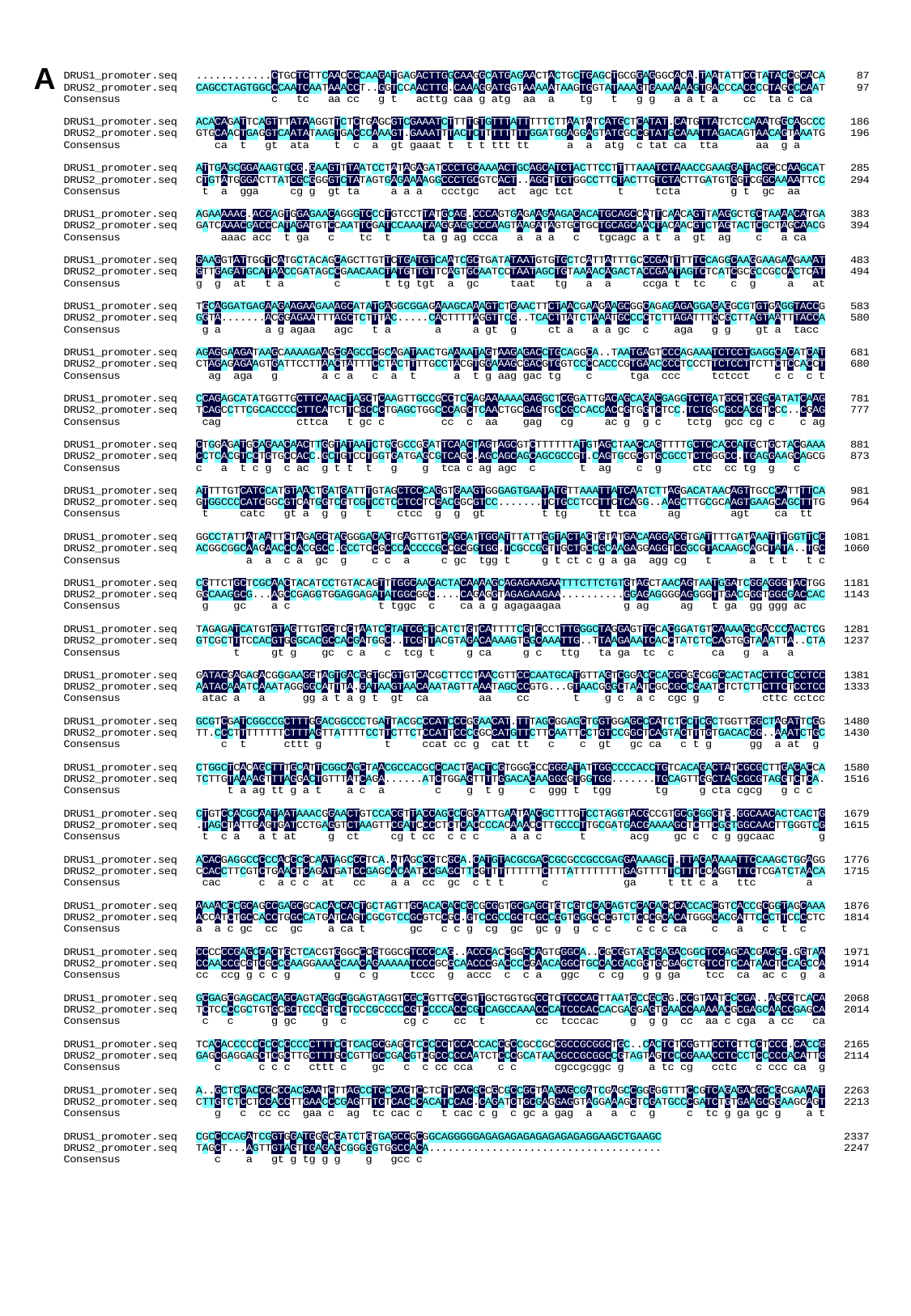

A

## Slide 10
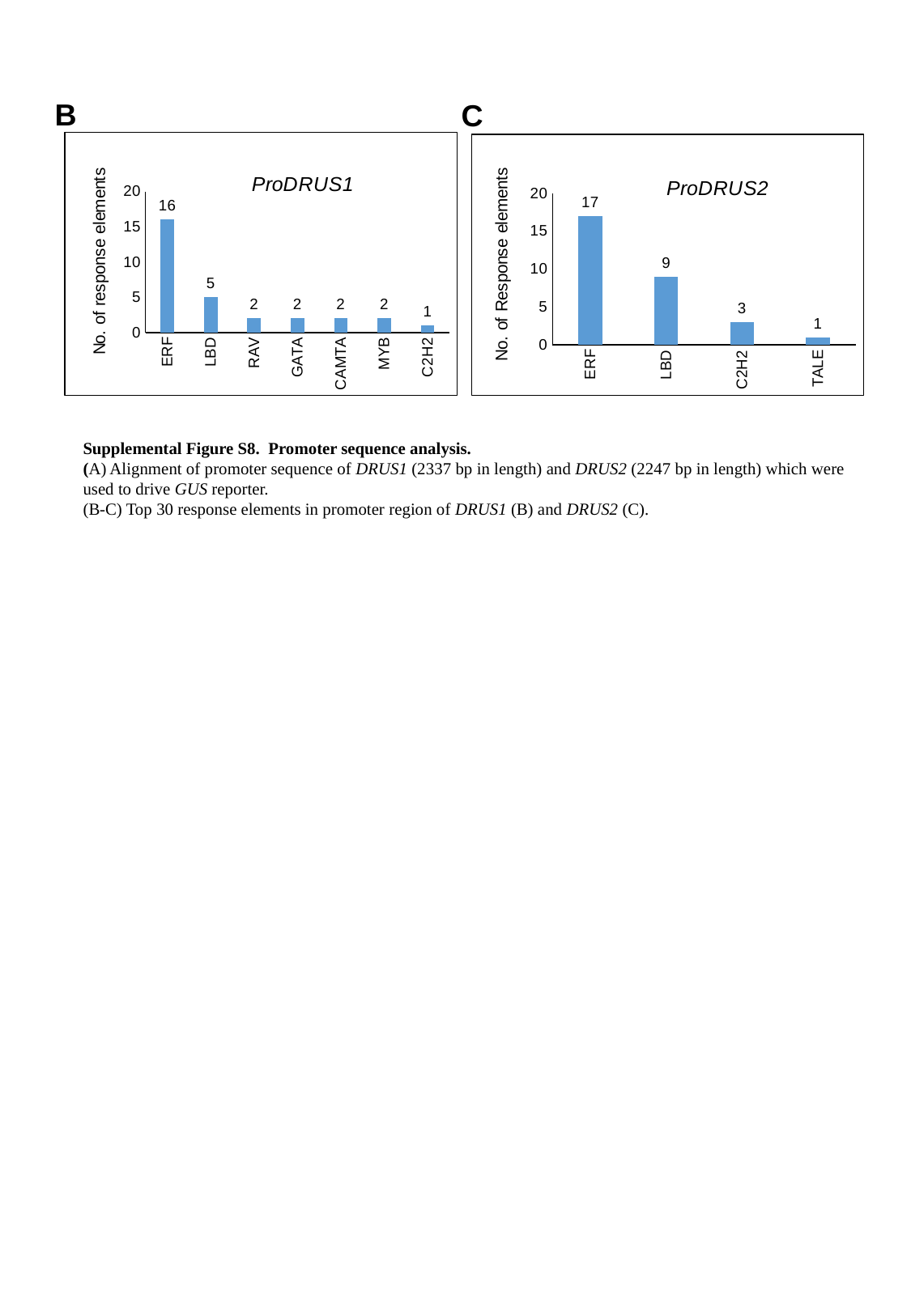

B
C
### Chart: ProDRUS1
| Category | |
|---|---|
| ERF | 16.0 |
| LBD | 5.0 |
| RAV | 2.0 |
| GATA | 2.0 |
| CAMTA | 2.0 |
| MYB | 2.0 |
| C2H2 | 1.0 |
### Chart: ProDRUS2
| Category | |
|---|---|
| ERF | 17.0 |
| LBD | 9.0 |
| C2H2 | 3.0 |
| TALE | 1.0 |Supplemental Figure S8. Promoter sequence analysis.
(A) Alignment of promoter sequence of DRUS1 (2337 bp in length) and DRUS2 (2247 bp in length) which were used to drive GUS reporter.
(B-C) Top 30 response elements in promoter region of DRUS1 (B) and DRUS2 (C).

## Slide 11
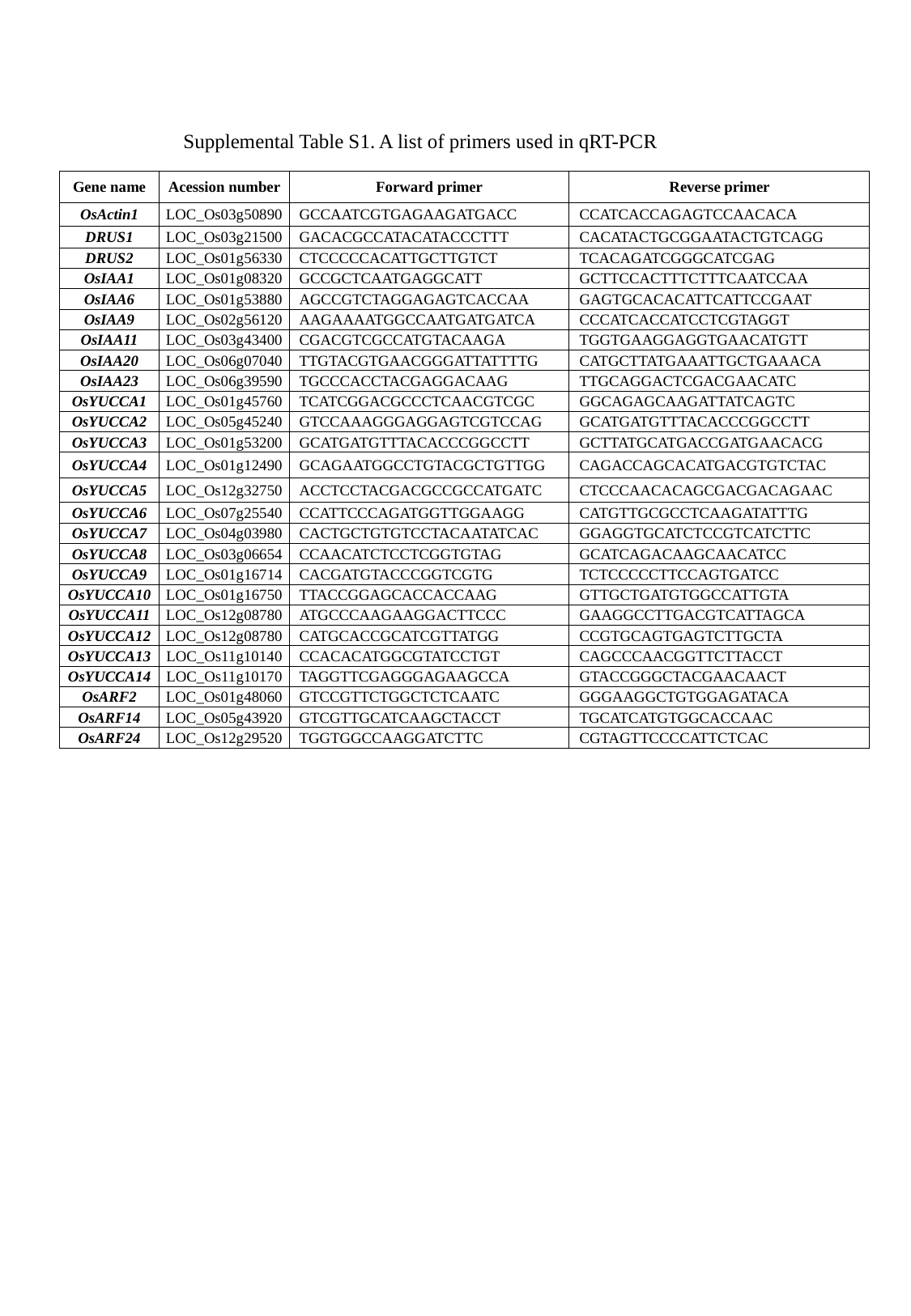

Supplemental Table S1. A list of primers used in qRT-PCR
| Gene name | Acession number | Forward primer | Reverse primer |
| --- | --- | --- | --- |
| OsActin1 | LOC\_Os03g50890 | GCCAATCGTGAGAAGATGACC | CCATCACCAGAGTCCAACACA |
| DRUS1 | LOC\_Os03g21500 | GACACGCCATACATACCCTTT | CACATACTGCGGAATACTGTCAGG |
| DRUS2 | LOC\_Os01g56330 | CTCCCCCACATTGCTTGTCT | TCACAGATCGGGCATCGAG |
| OsIAA1 | LOC\_Os01g08320 | GCCGCTCAATGAGGCATT | GCTTCCACTTTCTTTCAATCCAA |
| OsIAA6 | LOC\_Os01g53880 | AGCCGTCTAGGAGAGTCACCAA | GAGTGCACACATTCATTCCGAAT |
| OsIAA9 | LOC\_Os02g56120 | AAGAAAATGGCCAATGATGATCA | CCCATCACCATCCTCGTAGGT |
| OsIAA11 | LOC\_Os03g43400 | CGACGTCGCCATGTACAAGA | TGGTGAAGGAGGTGAACATGTT |
| OsIAA20 | LOC\_Os06g07040 | TTGTACGTGAACGGGATTATTTTG | CATGCTTATGAAATTGCTGAAACA |
| OsIAA23 | LOC\_Os06g39590 | TGCCCACCTACGAGGACAAG | TTGCAGGACTCGACGAACATC |
| OsYUCCA1 | LOC\_Os01g45760 | TCATCGGACGCCCTCAACGTCGC | GGCAGAGCAAGATTATCAGTC |
| OsYUCCA2 | LOC\_Os05g45240 | GTCCAAAGGGAGGAGTCGTCCAG | GCATGATGTTTACACCCGGCCTT |
| OsYUCCA3 | LOC\_Os01g53200 | GCATGATGTTTACACCCGGCCTT | GCTTATGCATGACCGATGAACACG |
| OsYUCCA4 | LOC\_Os01g12490 | GCAGAATGGCCTGTACGCTGTTGG | CAGACCAGCACATGACGTGTCTAC |
| OsYUCCA5 | LOC\_Os12g32750 | ACCTCCTACGACGCCGCCATGATC | CTCCCAACACAGCGACGACAGAAC |
| OsYUCCA6 | LOC\_Os07g25540 | CCATTCCCAGATGGTTGGAAGG | CATGTTGCGCCTCAAGATATTTG |
| OsYUCCA7 | LOC\_Os04g03980 | CACTGCTGTGTCCTACAATATCAC | GGAGGTGCATCTCCGTCATCTTC |
| OsYUCCA8 | LOC\_Os03g06654 | CCAACATCTCCTCGGTGTAG | GCATCAGACAAGCAACATCC |
| OsYUCCA9 | LOC\_Os01g16714 | CACGATGTACCCGGTCGTG | TCTCCCCCTTCCAGTGATCC |
| OsYUCCA10 | LOC\_Os01g16750 | TTACCGGAGCACCACCAAG | GTTGCTGATGTGGCCATTGTA |
| OsYUCCA11 | LOC\_Os12g08780 | ATGCCCAAGAAGGACTTCCC | GAAGGCCTTGACGTCATTAGCA |
| OsYUCCA12 | LOC\_Os12g08780 | CATGCACCGCATCGTTATGG | CCGTGCAGTGAGTCTTGCTA |
| OsYUCCA13 | LOC\_Os11g10140 | CCACACATGGCGTATCCTGT | CAGCCCAACGGTTCTTACCT |
| OsYUCCA14 | LOC\_Os11g10170 | TAGGTTCGAGGGAGAAGCCA | GTACCGGGCTACGAACAACT |
| OsARF2 | LOC\_Os01g48060 | GTCCGTTCTGGCTCTCAATC | GGGAAGGCTGTGGAGATACA |
| OsARF14 | LOC\_Os05g43920 | GTCGTTGCATCAAGCTACCT | TGCATCATGTGGCACCAAC |
| OsARF24 | LOC\_Os12g29520 | TGGTGGCCAAGGATCTTC | CGTAGTTCCCCATTCTCAC |
| | | | |
